# Enhancement of chilling stress tolerance in ornamental chilli by exogenous application of salicylic acid and ascorbic acid revealed through transcriptomic profiling

**DOI:** 10.3389/fpls.2026.1754279

**Published:** 2026-03-11

**Authors:** Anam Zahid, Fozia, Muhammad Ramzan, Gao Yike, Muhammad Mohsin, Salim A. Ali, Mohammad Fikry, Muhammad Munir, Hesham S. Ghazzawy, Hattim M. M. Makki, Khaled M. Ramadan, Mohamed A. A. Ahmed

**Affiliations:** 1 National Flower Engineering Research Centre, Beijing Key Laboratory of Ornamental Plants Germplasm Innovation and Molecular Breeding, College of Landscape Architecture of Beijing Forestry University, Beijing, China; 2 School of Forest Sciences, University of Eastern Finland, Joensuu, Finland; 3 Date Palm Research Center of Excellence, King Faisal University, Al-Ahsa, Saudi Arabia; 4 Central Laboratories, Department of Chemistry, King Faisal University, Al-Ahsa, Saudi Arabia; 5 Plant Production Department (Horticulture - Medicinal and Aromatic Plants), Faculty of Agriculture (Saba Basha), Alexandria Universitya, Alexandri, Egypt; 6 National Key Laboratory for Tropical Crop Breeding/Coconut Research Institute, Chinese Academy of Tropical Agricultural Sciences, Wenchang, Hainan, China

**Keywords:** ANK, ascorbic acid, *Capsicum annuum*, cobra, ERF, RNA-Seq, salicylic acid

## Abstract

Ornamental Chillies are valued for their potential to be marketed as potted plants, given their diversity in color and fruit shape. Despite their market value, Chillies are highly susceptible to chilling stress. Salicylic acid (SA) and ascorbic acid (AA) enhance plant cold tolerance by modulating antioxidant defense systems and stress-responsive signaling pathways. However, insufficient information exists on the overall gene expression induced by the combined foliar application of SA and AA under chilling stress in Chillies. In this study, RNA sequencing was utilized to elucidate the molecular mechanisms of combined SA+AA under chilling stress. A comprehensive transcriptomic analysis following exogenous treatment highlighted the impact of 2 mM SA and 2 mM AA on genes contributing to fundamental Chilling stress adaptation. The combined foliar application of SA+AA significantly reduced ROS accumulation by 48% in V1 and 54% in V2 compared with the control. Similarly, MDA content decreased to 3% in V1 relative to 9% and 8% in the control and V2, respectively, indicating reduced oxidative damage and enhanced cellular stability under chilling stress. There were 48346 gene transcripts, and within the sets, 210 genes were differently expressed (DEGs) following V1 treatment and 3933 genes following V2 treatment by SA and AA compared with the control. The cultivar WIZ-21 contains 123 Upstream and 77 downstream DEGs, and cultivar Golden Heart has 44 Upstream and 3979 downstream DEGs. Transcriptome analysis identified 48,346 expressed transcripts, of which a subset met predefined statistical thresholds and were classified as differentially expressed genes (DEGs). Six representative DEGs were selected for RT-qPCR validation of the RNA-seq results in both cultivars. The combined foliar treatment of SA and AA also stimulated the hormonal signaling in cytoplasm, and response to stress-related genes, such as COBRA, MYB14, MYC, GRP, ANK, and ERF proteins. The present investigation, therefore, pinpointed key genes that exhibited altered expression patterns in treated Chillies exposed to chilling stress, which were associated with hormone signaling and metabolism, redox, cellulose synthase-like proteins, and stress defense.

## 1 Introduction

Chilli belongs to the *Capsicum genus*, the most substantial genus in the *Solanaceae* family. Among them, *Capsicum annuum* is the most extensively grown and commercially valuable species. The high variability in fruit features makes the classification of Chilli crops challenging. For commercial purposes, Chilli goods are normally classified based on the differences in flavor, color, pungency, intended usage, as well as fruit shape and size (Azlan et al., 2022). Ornamental Chilli (*Capsicum annuum* L.) is valued due to its aesthetic appeal. These plants are edible and have attractive plant architecture, different fruit shapes, and varying levels of pungency. Compact growth (ideal for pots), a diverse range of unique fruit shapes and colors, and upright fruit orientation are all desirable features in ornamental Chilli (Aziza et al., 2024). Chilling stress is a familiar threat encountered by plants in climatically warm areas, which can dramatically impact yield. Although average global temperatures are rising, escalated occurrences of unseasonal frosts are affecting the potential distribution of thermophilic plant species (Sayed et al., 2024). Plants of a hot climate can be affected by low temperatures (0-12 °C) and can still be unfavorably injured by temperatures below 0 °C. When the temperature is less than 10 °C, plant growth slows, and there is a failure of photosynthetic and chlorophyll activity (Yang et al., 2020). The physical response of plants to chilling temperatures occurs in the plasma membrane. Their exposure to low temperatures alters the viscosity and structural integrity of membrane-associated proteins, metabolic disturbances, ionic imbalances, and, under extreme conditions, leads to cell degradation (Yadav, 2010; Guo et al., 2018). Plants have developed many adaptive approaches to survive in such circumstances. The initial reaction to cold is the accretion of reactive oxygen species (ROS), including OH·, O_2_·, and H_2_O_2_. To mitigate oxidative imbalance under Chilling stress, plants stimulate cellular detoxification response, including the greater production of antioxidant activity (Wang et al., 2017; Borella et al., 2023). Researchers have documented that chilling stress tolerance in plants includes broad remodeling of cellular structures, reprogramming of gene expression, and stimulating multiple protective mechanisms to respond to cold-induced damage. These include higher levels of intracellular solutes, addition of cryoprotectants and antioxidants, and the production of cold-regulated (COR) and anti-freeze proteins (Shukla et al., 2019; Zhang et al., 2022). Chillies, being a thermophilic crop, are highly susceptible to Chilling stress, which poses a significant challenge to their cultivation and development. Previous literature on chilling stress in Chillies has primarily highlighted physiological responses, transcription factors, and specific target genes (Gao et al., 2022; Hou et al., 2020). Wang et al. (2024) declared that exposure to Red LED light markedly enhances biomass quality by triggering an integrated signaling network that controls nitrogen uptake, supports amino acid production, and promotes protein formation in Chlorella pyrenoidosa. Transcription factors are key stimulators in Chilli’s defense against chilling stress; however, only an insufficient TF family, likely as bHLH, MADS, and NAC, have been known as supporters to cold tolerance in Chillies (Chen et al., 2020; Hou et al., 2020; Zhang et al., 2020, 2021). To protect cellular integrity and maintain normal functions under Chilling stress, plants have developed various adaptive strategies. Among these, the CBF (C-Repeat Binding Factors), ICE (Inducer of CBF Expression), and COR (Cold-Regulated) signaling cascades are the most important pathways involved in the defense response against Chilling stress (Wang et al., 2017; Shi et al., 2017). Recent developments in transcriptomic analysis have revealed that a higher number of TIFY family genes participate in Chilling stress responses (Zhang et al., 2012, 2015, 2022; He et al., 2020). Numerous BdTIFY genes are activated by environmental stresses, including salinity, cold, drought, and heat (Zhang et al., 2015). Additionally, Zhao et al. (2019) observed that in wheat, Chilling stress significantly enriched DEGs and metabolites DEMs linked with proline biosynthesis and abscisic acid/jasmonic acid (ABA/JA) signaling pathways.

Plant growth regulators (PGRs) are compounds that can improve plant development, sustain yields, enhance crop productivity and quality, and strengthen tolerance to environmental stresses (Yirmibe et al., 2025). PGPR have emerged as key contributors to sustainable crop production. Through improved nutrient dynamics, growth regulation, and stress alleviation, these beneficial microbes support plant adaptation to challenging environmental conditions while enhancing agricultural productivity (Gharbi et al., 2025). Furthermore, salicylic acid (SA) is a particularly important hormone in Chilling stress responses. SA is produced via two main pathways: from isochorismic acid via the isochorismate pathway, or from benzoic acid along the phenylalanine ammonium lyase pathway (Saleem et al., 2021).

Different plant species have different influences of SA on the expression of CBF genes. For example, SA has been documented to decrease frost injury in peach reproductive organs and improve frost resistance by enhancing CBF gene activation (Zhang et al., 2017). Similarly, Wang et al. (2021) declared that SA has only a minor, non-significant impact on CBF gene activity in wheat.

Ascorbic acid (AA) promotes photosynthesis in rice by maintaining chlorophyll content, stabilizing photosynthetic membranes, and controlling photosynthetic enzyme activity (Feng et al., 2020). In Arabidopsis thaliana, AA-deficient mutants exhibited early blooming under long-day conditions (Kotchoni et al., 2009). Under abiotic stress circumstances, AA is an effective non-enzymatic antioxidant that is essential for scavenging free radicals and reducing oxidative stress in plants (Majzoobi et al., 2014; Gęgotek and Skrzydlewska, 2023).

Exogenous application of AA improves tomato and rice seed germination under low temperature stress by accumulating soluble sugar and CAT, POD, APX, SOD, amylase, and α-amylase activities (Elkelish et al., 2020; Monajjem et al., 2023). Similarly, elevated expression of *PbDHAR2* from *Pyrus sinkiangensis* in tomato improves AA accumulation and enhances tolerance to chilling and salinity stress (Qin et al., 2015). Li et al. (2025) declared that the Combined application of Phytohormone with nitrogen enhances the lutein production in the Chlorella protothecoides during heterotrophic cultivation.

Beyond their developmental activities, developing evidence specifies that COBL genes play focal roles in plant responses to climatic stresses such as cold, salinity, heat, and drought. Comparative genomic investigations in *Arabidopsis, Populus*, and *Zea mays* have revealed that various COBL family members display distinctive expression patterns and contribute to improved stress adaptation across different environmental circumstances (Ye et al., 2009; Zaheer et al., 2022; Li et al., 2022). Five TaCOBLs were markedly downstream under dehydration stress in wheat (Zaheer et al., 2022). The apple MYB transcription factor MdMYB308L promotes cold tolerance and anthocyanin production by forming a complex with MdbHLH33, thereby facilitating its binding to the upstream sequence of MdDFR and MdCBF2 (An et al., 2020). In *Arabidopsis*, overexpression of the crabapple MYB4 gene considerably enhances the resistance to drought and Chilling stress (Yao et al., 2022a). Generally, numerous investigations confirm that the MYB TF family plays a fundamental function in regulating chilling stress responses and providing chilling resistance (Wu et al., 2021; Dar et al., 2022; Yao et al., 2022b).

MYC gene overexpression enhanced frost susceptibility and inhibited the transcription of genes that respond to cold. The MYC genes attach to the promoter elements in the CBF3/DREB1A, likely interfering with ICE1’s ability to interact with these cis elements (Ohta et al., 2018). The transcription factor APETALA2/ethylene response factor (AP2/ERF) is crucial for modulating the expression of genes associated with environmental stress (Du et al., 2016; Cao et al., 2019). Therefore, ERF102–ERF105 are like other ERF transcription factors that serve as central regulatory hubs, integrating hormonal signaling in plant responses to environmental stresses (Illgen et al., 2020). Ornamental chilli (*Capsicum annuum* L.) is a commercially important horticultural species widely used in ornamental landscaping and container production. Despite its high market value, the crop is particularly sensitive to chilling stress that adversely affects plant growth and aesthetic quality. Therefore, it serves as a suitable system for studying physiological approaches to improve chilling tolerance and production stability. To bridge this knowledge gap, we examined the physiological and transcriptomic responses of ornamental Chilli plants to foliar application of SA and AA bio-stimulants under Chilling stress. Foliar application of bio-stimulant was evaluated for its impact on morphological and physiological traits. In addition, at the molecular level, RNA-Seq analysis was performed to unravel the gene expression profiles modulated by SA and AA treatment, thereby identifying key molecular pathways and targets associated with stress tolerance. In general, our study offers significant perspectives for developing reliable approaches to improve ornamental Chilli productivity under Chilling stress.

## 2 Materials and methods

### 2.1 Plant material of transcriptomic analysis

**Figure 1:**
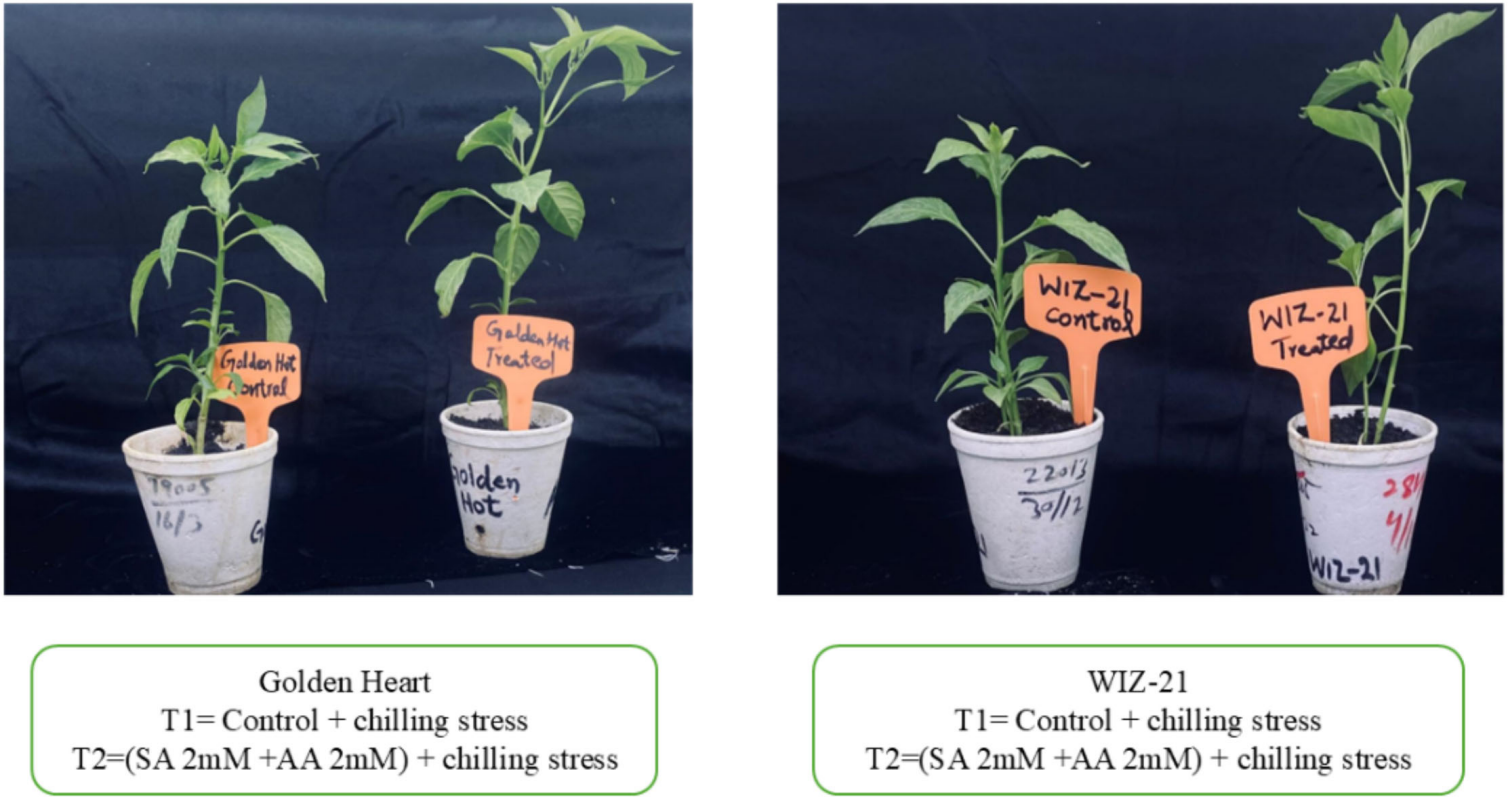
Morphological changes in chill-stressed ornamental Chilli seedlings with or without exogenous application of SA+AA in both cultivars under chilling stress conditions.

The annual plants of ornamental Chillies WIZ-21 and Golden Heart varieties were used in the research under Chilling stress. Chilli seeds were grown in a 50-cell plug tray filled with a mixed substrate comprising peat, vermiculite, and perlite with the proportion of 2:1:1 (v/v/v). The seedlings were raised in a greenhouse under controlled-environment day/night temperatures of 25 °C (16 h light) and 20 °C (8 h dark) until the stage of four true leaves. Uniformly growing seedlings were screened and relocated into 9 cm × 9 cm pots. The relative humidity was between 60% and 80%. Seedlings were maintained until the six-leaf stage was reached and divided into two groups: one was foliar sprayed with combined Salicylic acid and Ascorbic acid (SA 2 mM + AA 2 mM), and the other was sprayed with distilled water and then subsequently exposed to Chilling stress at 6 °C for 48 hours. The selection of the dose was based on the previous study (Zahid et al., 2023). The (SA 2 mM + AA 2 mM) solution was identified as the most effective dose for pretreating ornamental Chilli seedlings, while the control was treated with distilled water. The Chilli seedlings were sprayed once. Leaf samples were harvested for further Phenotypic indexes, and transcriptomic experiments were performed with three independent biological replicates per treatment. The samples were preserved by freezing in liquid nitrogen and kept at −80 °C for RNA-Seq analysis. The samples were labeled with chilling stress + sprayed (SA 2 mM + AA 2 mM) (T2) and chilling stress+ control (T1) (**Figure 1**).

### 2.2 Phenotypic and growth parameters

For the measurement of shoot and root length, plants were carefully uprooted from the pots, and roots were thoroughly rinsed with distilled water to detach adhering substrate. Shoot and root lengths were thereafter measured using a calibrated ruler. A digital weighing balance measured the fresh biomass of the roots and the shoots.

### 2.3 Physiological analyses of ornamental Chillies

Lipid peroxidation in leaf tissues was determined by measuring malondialdehyde (MDA) accumulation as described by Cakmak and Horst (1991) with slight changes. Freshly harvested leaf tissue (0.2 g) was crushed in a chilled mortar and pestle, then homogenized in 1 mL of 0.1% trichloroacetic acid (TCA). The tissue extract was centrifuged at 2,000 × g for 15 min, and 0.5 mL of the centrifuged fraction was homogenized with 1 mL of 0.5% thiobarbituric acid (TBA) in 20% TCA. The prepared mixture was heated at 95 °C for 50 min in a shaking water bath and rapidly placed on ice to terminate the reaction. After centrifugation at 12,000 × g for 10 min, the absorbance of the supernatant was estimated at 532 and 600 nm. MDA levels were measured, indicating lipid peroxidation, by using the standard formula.

Malondialdehyde (MDA) = Δ (A 532 nm−A 600 nm)/1.56×105.

Hydrogen peroxide (H2O2) concentration was estimated with the procedure of Velikova et al. (2000). A 0.2 g leaf sample was powdered with the addition of 2 mL of 0.1% TCA, and the obtained tissue extract was centrifuged at 6,000 × g for 15 min. Subsequently, 100 µL of the resulting supernatant was mixed with 100 µL of 50 mM phosphate buffer (pH 7.2) and 200 µL of 1 M potassium iodide. The absorbance of the reaction mixture was noted at 390 nm, assessed via spectrophotometry, and H_2_O_2_ concentration was quantified based on a calibration curve.

### 2.4 RNA Extraction and RNA-seq

Leaf RNA was isolated from the control and sprayed samples Trizol reagent (Invitrogen, Carlsbad, CA, USA) according to the technique demonstrated by Hu et al. (2012). The extracted RNA was separated into two aliquots: one allocated for RNA sequencing, and the other for quantitative real-time PCR (qRT-PCR). RNA-seq analysis was accomplished with an Illumina HiSeq 4000 platform (Illumina, San Diego, CA, USA) at Alpha Genomics Bioinformatics Co., Pakistan. Raw sequencing reads were trimmed and filtered out low-quality reads with adaptor sequences or ambiguous nucleotides, and high-quality reads were generated by the procedure of Chen et al. (2014). Filtered reads were smoothly mapped to the reference genome of Capsicum annuum assembly UCD10Xv1.1 - NCBI - NLM (NIH, US, 2025).

### 2.5 Differentially expressed analysis

Differential expression analysis between the control and Chilling+(SA 2 mM + AA 2 mM) treatments was undertaken following the DESeq package in R (Wang et al., 2010), which employs a negative binomial distribution model for statistical assessment of gene expression counts. To regulate the false discovery rate (FDR), we adjusted the obtained p-values (Benjamini and Hochberg, 1995), and different DEGs were identified according to the FDR-adjusted p-value **<** 0.05. We completed gene ontology (GO) functional annotation analysis of the DEGs corresponding to the GOseq R package and GO terms with q less than 0.05 were considered as suggestively enriched (Young et al., 2010). The functional enrichment of DEGs in Kyoto Encyclopedia of Genes and Genomes (KEGG) pathways was performed with KOBAS software (Xie et al., 2011). Consequently, differentially expressed genes between control samples and chilling-sprayed with SA and AA were acknowledged by means of DESeq2 software, with |log2 fold change (FC)| greater than 1 and FDR.

### 2.6 Validation of genes by real-time quantitative PCR

Six candidate genes were selected for qRT-PCR quantification; the selected genes and specific primers are listed in the **Table 1**. GAPDH was utilized as a housekeeping gene. Real-time quantitative PCR was performed using SYBR-Green (ABI-Invitrogen, California, USA) on an ABI 7900 Fast Real-Time PCR Detection System (Applied Biosystems, Carlsbad, USA). Each 20 µl reaction was formulated with 10 µl of 2× SuperReal PreMix Plus, 2 µl cDNA, 1 µl of each primer, and 6 µl nuclease-free water, and subjected to 40 cycles of amplification. Relative expression of 6 target genes, standardized to GAPDH (reference gene), was estimated from Ct values using the 2-ΔΔCt method (Livak and Schmittgen, 2001).

**Table 1:** Primers sequence for qRT-PCR.

Genes	LocusID	Forward primer (5′→3′)	Reverse primer (5′→3′)	Product size (bp)
MYB14	LOC107872700	GGCCTTAAGTAATTTATCCACC	CCATGGACTCAAGAAGAAGA	143
ERF	LOC107851517	ACATATTCCTCCTCATCCCT	GATGATGATCGGTTGTTGGT	140
MYC1	LOC107850747	ATCACTCAGATCTCGAGGC	AATTTCTCCCTCCTCTGTCT	145
GRP	LOC107840409	CATGAAGGATGCTATTGAAGGGA	CTTCACGTCGGCCACCT	140
ITN1	LOC107853651	CATGTCAGGTTCCTCACAC	TGAAATCTTGAACTCCTGCA	141
COBRA	LOC124893127	GATATACATGTGGTCAGCCC	CTGCATATGGAGCATGGTAC	145
Housekeeping gene	GAPDH	GCACCTATGTTTGTGGTTGGAGTA	ACCGTCTTCTGTGTAGCTGTTGTT	138

### 2.7 Statistical analysis

Chemical and qRT-PCR data were assessed by one-way analysis of variance (ANOVA) in SPSS software (version 16.0; SPSS Inc., Chicago, IL, USA). All experiments were conducted with three independent biological replicates, and the results are presented as mean ± standard error (SE). Differences were deemed statistically significant at p < 0.05.

## 3 Results

### 3.1 Morphological traits

The Foliar application of SA and AA in combination (T2) treatment markedly improved the early growth performance of Chilli seedlings compared to the control (T1). Statistically significant differences *(p < 0.05)* were noted across all measured traits, with considerable varietal changes in the magnitude of response. Seedling length was significantly enhanced under T2 treatment in both varieties (V1 and V2). In WIZ-21, seedling length nearly doubled compared to the non-treated, whereas Golden heart also exhibited a significant enhancement, though less pronounced than WIZ-21. Statistical grouping letters reveal that T2 was significantly different from the control treatment (T1) within both varieties, confirming the growth-enhancing role of bio-stimulants (SA + AA). Shoot elongation responded greatly to the combined SA + AA application. Plants under T2 presented notably greater shoot length (18.33 cm) than their respective controls (8.33 cm). The effect was particularly prominent in WIZ-21, where shoot length was enhanced by two-fold as compared to the control, whereas Golden heart showed a moderate but still statistically significant improvement. These results suggest that the combination of SA and AA effectively prompted shoot vigor and photosynthetic tissue development.

Root development was also completely influenced by T2. Root length was detrimentally enhanced in both varieties, with V1 (9.0 cm) revealing a greater stimulatory response than V2 (8.0 cm). Statistically significant differences between treatments confirmed that the SA + AA combination improved root architecture, potentially strengthening the plants’ ability to acquire water and nutrients. Consistent with root elongation, root biomass accumulation was significantly higher under combined SA+AA treatment as compared to control plants. Both varieties showed positive responses to the treatment, as V2 produced a root fresh weight (0.4 g/p) greater than V1, inferring varietal differences in biomass allocation. This indicates that while V1 exhibited a stronger response in elongation traits, V2 invested more resources into root mass development.

**Figure 2:**
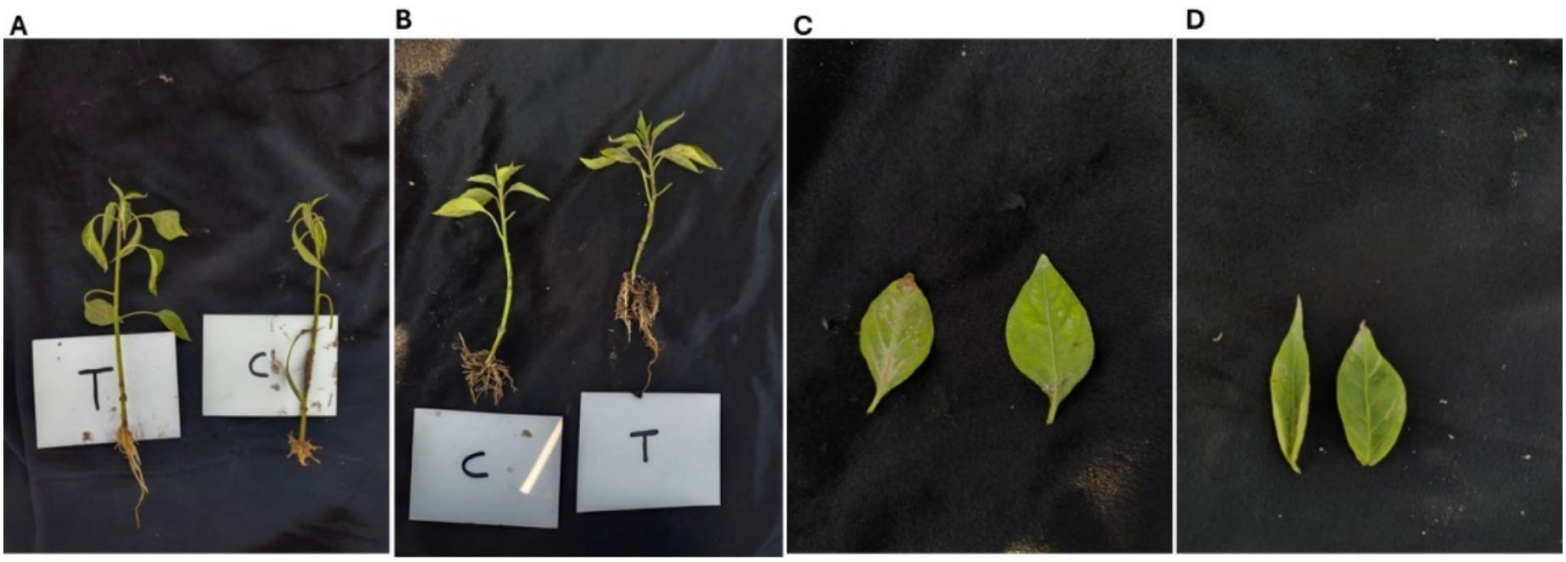
Morphological traits of WIZ-21 and Golden Heart varieties **(A)** Representative seedling of WIZ-21 showing shoot and root length. **(B)** Representative seedling of Golden Heart showing shoot and root length. **(C)** Leaves of WIZ-21 illustrating leaf length. **(D)** Leaves of Golden Heart illustrating leaf length.

Collectively, the results explained that the combined treatment with SA and AA significantly enhanced both root and shoot growth parameters compared with the untreated control. Variety V1 exhibited greater improvements in elongation traits such as seedling and shoot length, whereas V2 responded more strongly in terms of root biomass accumulation. These findings reveal that the combined foliar spray of SA and AA can serve as an effective growth-promoting strategy in ornamental Chilli plants, potentially by acting synergistically to improve physiological performance, regulate metabolic processes, and strengthen antioxidant defenses during the preliminary phase of seedling growth. The combined foliar spray of SA and AA T2 treatment had a positive influence on seedling growth compared to the untreated control (T1), with consistent positive effects across leaf and root parameters, and clear varietal differences in the extent of response (**Figure 2**).

Leaf length was increased significantly in the treated plants in both Chilli varieties. V1 showed the 64% most pronounced effect, with leaves almost doubling in length compared with non-treated plants, whereas Golden heart also exhibited a statistically significant, yet relatively lower, improvement. The expansion in leaf elongation under T2 indicates that the combination of SA and AA may perform synergistically to promote leaf growth and cell expansion, thereby influencing the overall vigor of the Chilli seedlings (**Figure 2**).

Root width was effectively elevated by the combined spray of SA + AA. In WIZ-21, the root width under the T2 treatment increased by 34.6% compared with the T1 treatment, demonstrating a clear stimulatory effect on root development. While Golden Heart presented a lower response, it still exposed a significant improvement over the control. These findings suggest that combined foliar application of SA and AA enhances below-ground growth, likely by stimulating root initiation and lateral root development, thereby improving resource uptake and plant anchorage.

**Figure 3:**
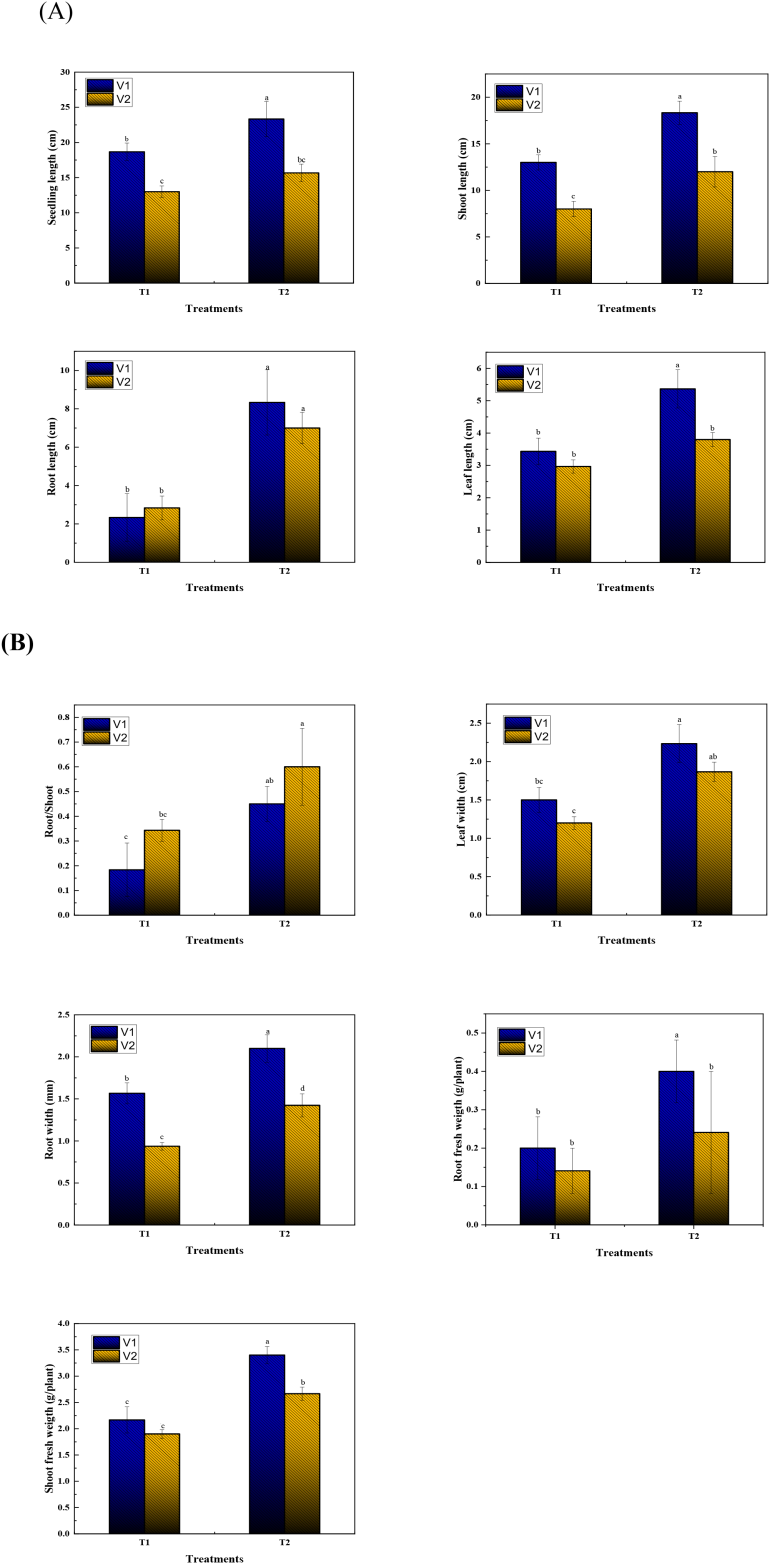
Effect of SA and AA on the growth of Chilli seedlings under Chilling stress. **(A)** Ornamental Chilli seedling phenotype, shoot length, root length, leaf length, and seedling length **(B)** leaf width, root width, root fresh weight, root/shoot, and shoot fresh weight. Different letters specify a significant difference between mean values at P < 0.05. T1: Chilli seedlings treated with simple water at chilling temperature; T2: (SA 2 mM + AA 2 mM) was foliar sprayed at low temperature.

A notable increase in leaf area was assessed under T2 in both varieties, such as WIZ-21 and Golden Heart. V1 exhibited the most striking response, with leaf area doubled compared to non-sprayed plants, whereas Golden Heart also proved a significant though comparatively smaller expansion. The Enlargement of leaf area under a combination of SA+AA recommends an increase in photosynthetic surface, which is vital for maintaining higher metabolic activity and supporting root and shoot growth instantaneously (**Figure 3A**).

The accumulation of root dry matter was substantially higher under combined SA+AA compared to the control, verifying that combined SA and AA foliar applications provide not only root volume and elongation but also biomass allocation toward root tissues. WIZ-21 showed a greater response than Golden Heart, developing heavier root systems. This study suggests that SA and AA may promote efficient allocation of acclimatization toward root growth, mostly enhancing plant robustness and sustained growth capacity.

Together, these results show that combined foliar application of SA and AA (T2) significantly increases both leaf length, leaf area, root volume, and root dry weight traits of Chilli seedlings compared with untreated plants (T1). The consequences were consistently higher in V1, particularly in elongation and leaf attributes, whereas V2 showed moderate to significant improvements. This varietal comparison suggests that genetic background may limit the extent to which plants are supported by combined SA and AA supplementation. The results emphasize that the combined foliar applications of SA and AA act as a growth-promoting approach, theoretically through ameliorated cell expansion and division, improvement of photosynthetic surface area, inducement of root biomass, and promoting balance of shoot–root growth dynamics.

The combined application of T2 (SA+AA) had a significant effect on the oxidative stress signal in ornamental Chilli plants compared with the control (T1). The results revealed a distinct reduction in reactive oxygen species generation and membrane lipid peroxidation in seedlings treated with SA + AA, with prominent varietal differences in the extent of the response. (**Figure 3B**).

### 3.2 Physiological analyses of ornamental Chillies

**Figure 4:**
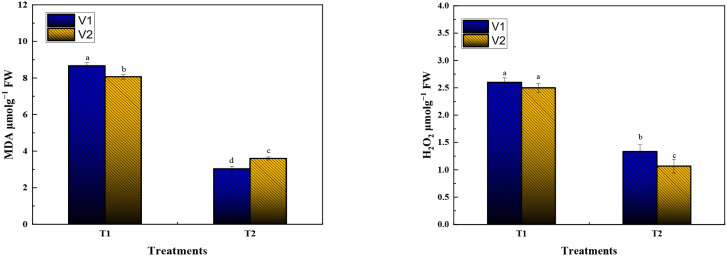
Effect of SA and AA on the growth of Chilli seedlings under Chilling stress. H_2_O_2,_ and MDA. Different letters specify a significant difference between mean values at P < 0.05. T1: Chilli seedlings treated with simple water at chilling temperature; T2: (SA 2 mM + AA 2 mM) was foliar sprayed at low temperature.

A substantial decrease in hydrogen peroxide content was noted in combined SA+AA-treated plants across both varieties. Under T1, the hydrogen peroxide (H_2_O_2_) concentration was approximately 3.2 µmol g^−1^ FW in V1 and 3.0 µmol g^−1^ FW in V2, with no statistically significant variation between the two varieties. In contrast, under T2, the H_2_O_2_ levels declined noticeably, reaching around 1.9 µmol g^−1^ FW in V1 and 1.6 µmol g^−1^ FW in V2. In WIZ-21, (H_2_O_2_) contents were mitigated more pronouncedly than in V2, reflecting an elevated antioxidative capacity. The diminished accumulation of H_2_O_2_ in T2 reveals that the combined SA + AA treatment efficiently regulated ROS homeostasis, owing to suppressing overproduction at the cellular level or augmenting the function of enzymatic and non-enzymatic antioxidant defenses (**Figure 4**).

H_2_O_2_ concentration resulted in a parallel decline under combined SA+AA treatment. In Comparison with Control, plants subjected to bio-stimulant treatment presented significantly lower concentrations of H_2_O_2_ in both varieties, with WIZ-21 again displaying an enhanced response. As a result, H_2_O_2_ maintained stable ROS levels and often functions as a molecular signal; its regulated drop reflects that the combined foliar application of SA and AA not only mitigated oxidative stress but also sustained a homeostatic balance between ROS signaling and detoxification. This decrease is possibly associated with the stimulated activity of catalases, peroxidases, and ascorbate–glutathione cycle components, which collectively mitigate H_2_O_2_ levels (**Figure 4**).

Malondialdehyde (MDA), a lipid peroxidation derivative, was considerably reduced under T2 as compared to T1. The pronounced decrease in MDA concentration demonstrates that combined SA and AA co-treatment maintained membrane stability by curbing ROS-triggered lipid peroxidation of polyunsaturated fatty acids. WIZ-21 seedlings revealed the minimal MDA levels under T2 (4.0 µmol g^−1^ FW), consistent with more effective protection of membrane integrity under oxidative stress relative to Golden Heart (3.0 µmol g^−1^ FW). These outcomes are in accordance with the observed decreases in H_2_O_2_. supporting the contribution of combined SA and AA in alleviating cellular membranes, which is mitigated by oxidative stress damage (**Figure 4**).

In aggregate, the attenuation in H_2_O_2_ and MDA concentrations in T2 elaborates the potency of SA and AA combined co-application in relieving oxidative stress in ornamental Chilli seedlings. By minimizing ROS enrichment and suppressing lipid peroxidation, the treatment facilitates the maintenance of cellular homeostasis and preserves structural and functional integrity. The influential antioxidative activity in WIZ-21, as compared to Golden Heart, emphasizes probable genotypic differences in the competence to harness SA and AA for stress alleviation. These results recommend that the SA + AA combination stimulates not only growth improvement but also physiological resistance, implemented through enhanced redox regulation, cellular membrane stability, and antioxidant activity (**Figure 4**).

### 3.3 Differentially expressed genes and analysis of expression pattern

In transcriptomic studies, Venn diagrams serve as an effective tool for visualizing the overlap and divergence of differentially expressed genes (DEGs) across experimental conditions, treatments, or genotypes. By representing shared and unique gene sets, Venn diagrams provide a straightforward means of assessing the extent of transcriptional similarity or specificity among biological samples. For instance, genes present in the intersection highlight core transcriptional responses that are conserved across groups, whereas unique gene sets reveal genotype- or condition-specific regulatory patterns. RNA-seq-based differential expression analysis was conducted with a significant criterion of |log_2_ (fold change) | ≥ 1 in combination with a p-value threshold to ensure comprehensive profiling of stress-responsive genes. Pairwise comparisons between sprayed (SA+AA) and control plants under Chilling stress were employed to identify the differentially expressed genes (DEGs). The number of DEGs was influenced by the type of stress, variety, and treatment. For each experiment, DEGs were further categorized into upregulated or downregulated groups for each treatment across both varieties. The number of differentially expressed genes identified in each variety under low temperature stress indicated substantial variation in transcriptional responses.

**Figure 5:**
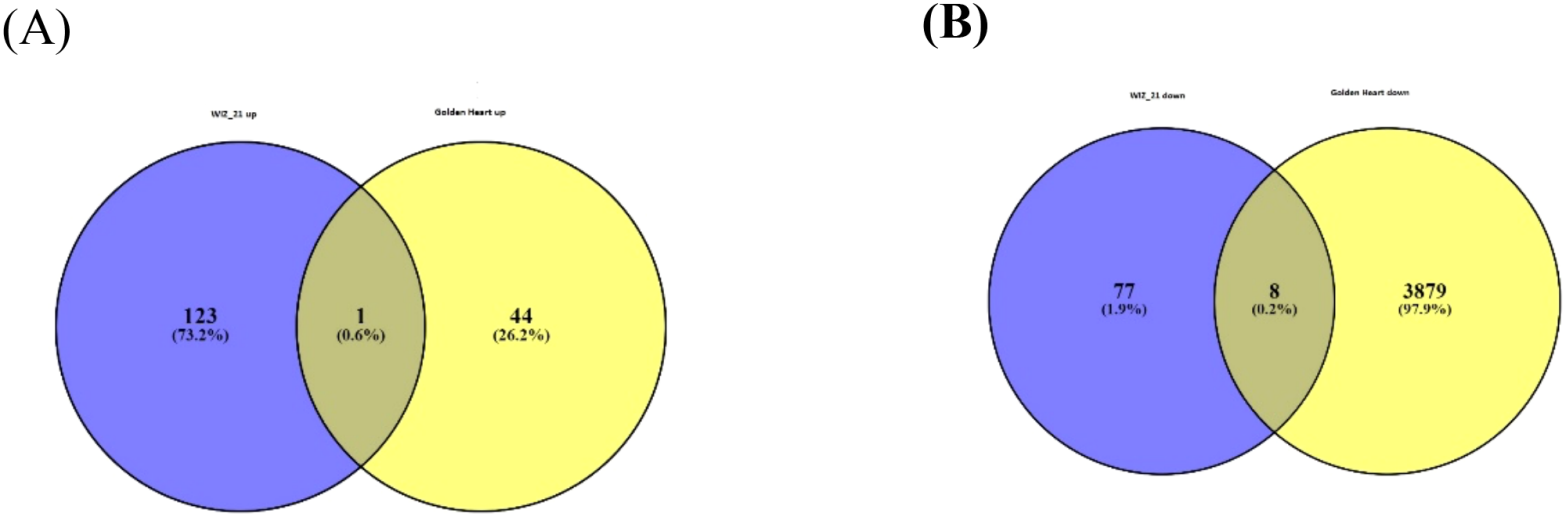
Effect of foliar application of SA and AA on Gene expression profile of different Chilli cultivars in response to Chilling stress. **(A)** Venn diagram of Upstream DEGs. **(B)** Venn diagram of downstream DEGs in both cultivars.

In the Wiz-21 variety, 209 DEGs were triggered by Chilling stress. Among these, 123 genes were overexpressed, indicating activation of stress-signaling pathways, although 77 genes were negatively regulated, indicating preferential inhibition of pathways. The up-regulated genes might involve primary factors in defense pathways, such as participating in antioxidant defense, osmotic adjustment, signaling cascades, and transcriptional regulation, synergistically maintaining cellular homeostasis during Chilling stress. The down-regulated genes, although representing a smaller proportion, may specify targeted suppression of growth-related or non-essential metabolic pathways, thus allowing the plant to divert energy and inputs toward stress survival. This transcriptional profile suggests that WIZ-21 predominantly mitigates Chilling stress through transcriptional activation, with a higher proportion of genes exhibiting induced expression (**Figure 5**). By comparison, Golden Heart manifested a markedly varied transcriptional response, with 3,923 DEGs annotated under Chilling stress. Remarkably, only 44 genes were upstream, in contrast to the predominant 3,879 genes that were downstream. This striking imbalance highlights a global transcriptional repression, implying that Golden Heart mainly responds to Chilling stress by repressing a range of cellular functions rather than actively contributing to stress-defensive pathways. The widespread downregulation of genes may be considered an energy-conservation approach under unfavorable conditions, theoretically limiting growth and metabolic adaptability. The limited number of up-regulated genes suggests a minimal activation of responsive or protective mechanisms, possibly making Golden Heart more prone to cold exposure (**Figure 5**).

**Figure 6:**
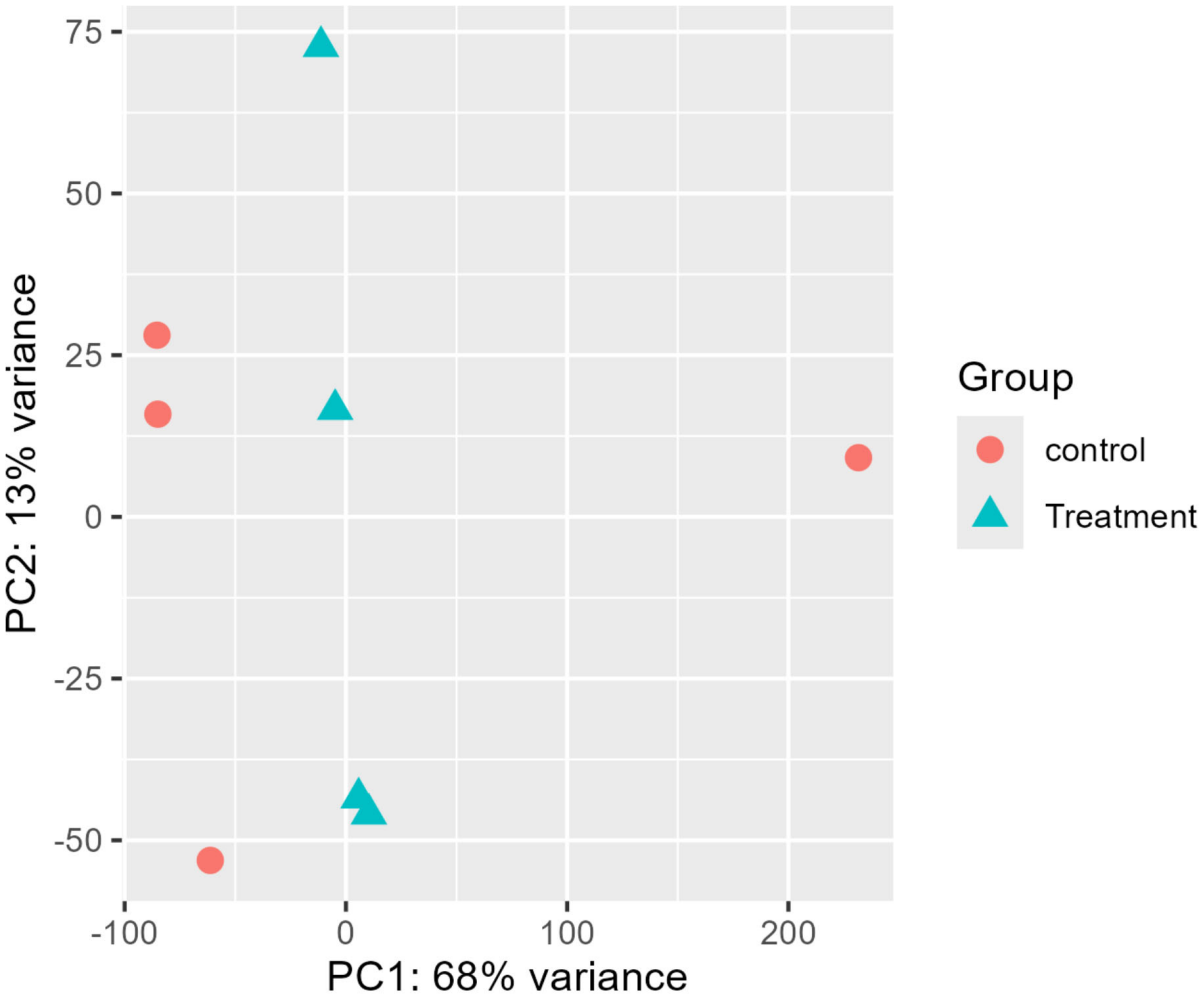
Principal component analysis (PCA) shows the effect of Foliar treatment of SA and AA on DEGs of Chilli leaves under Chilling stress.

To elucidate the transcriptional reprogramming underlying plant acclimation to Chilling stress and applied treatments, a Principal Component Analysis (PCA) was carried out on the gene expression database. This multivariate statistical approach facilitated the reduction of complex, high-dimensional gene expression datasets into principal components (PC), which in turn aided in identifying the main sources of variation. PCA was conducted to visualize the dataset’s overall variability and to investigate whether the control and treatment groups exhibit distinct clustering patterns. The first two principal components accounted for 81% of the total variance, with PC1 explaining 68% and PC2 explaining 13%. The PCA score plot revealed distinct clustering between control and treated samples along PC1, indicating a strong treatment-driven effect on global gene expression patterns (**Figure 6**). Control samples were tightly grouped along the negative axis of PC1, reflecting a relatively homogeneous transcriptional profile. In contrast, treated samples were positioned closer to the origin but displayed greater dispersion along PC2, suggesting increased transcriptional heterogeneity in response to treatment. Conversely, samples subjected to treatment were projected primarily near the origin along PC1; however, they exhibited higher variability along PC2. This trend points to the fact that while it clearly distinguishes the treated samples from the non-treated group, in addition, treated plants exhibited higher heterogeneity, which could be due to varying physiological or molecular responses among treated samples. Notably, a single control replicate appeared marginally projected towards the positive PC1 axis, suggesting limited within-group variation that may be caused by factors arising from biological or technical. The overall distinction between control and treated plants further indicates that exposure to treatment resulted in notable physiological modifications in the plant system. (**Figure 6**).

### 3.4 Effects of SA+AA on differentially expressed genes analysis in the chilli leaves

Volcano plots provide a fundamental graphical method for emphasizing differentially expressed genes in RNA-seq analyses. The volcano plot displays the DEG profiles for the two varieties, WiZ-21 and Golden Heart, with the x-axis demonstrating the log_2_ fold change and the y-axis indicating the -log_10_ p-value for each transcript or feature. In both plots, the data points are color-coded based on statistical significance: red dots indicate significantly elevated gene expression, green points indicate reduced gene expression, and black represents non-significant features.

**Figure 7:**
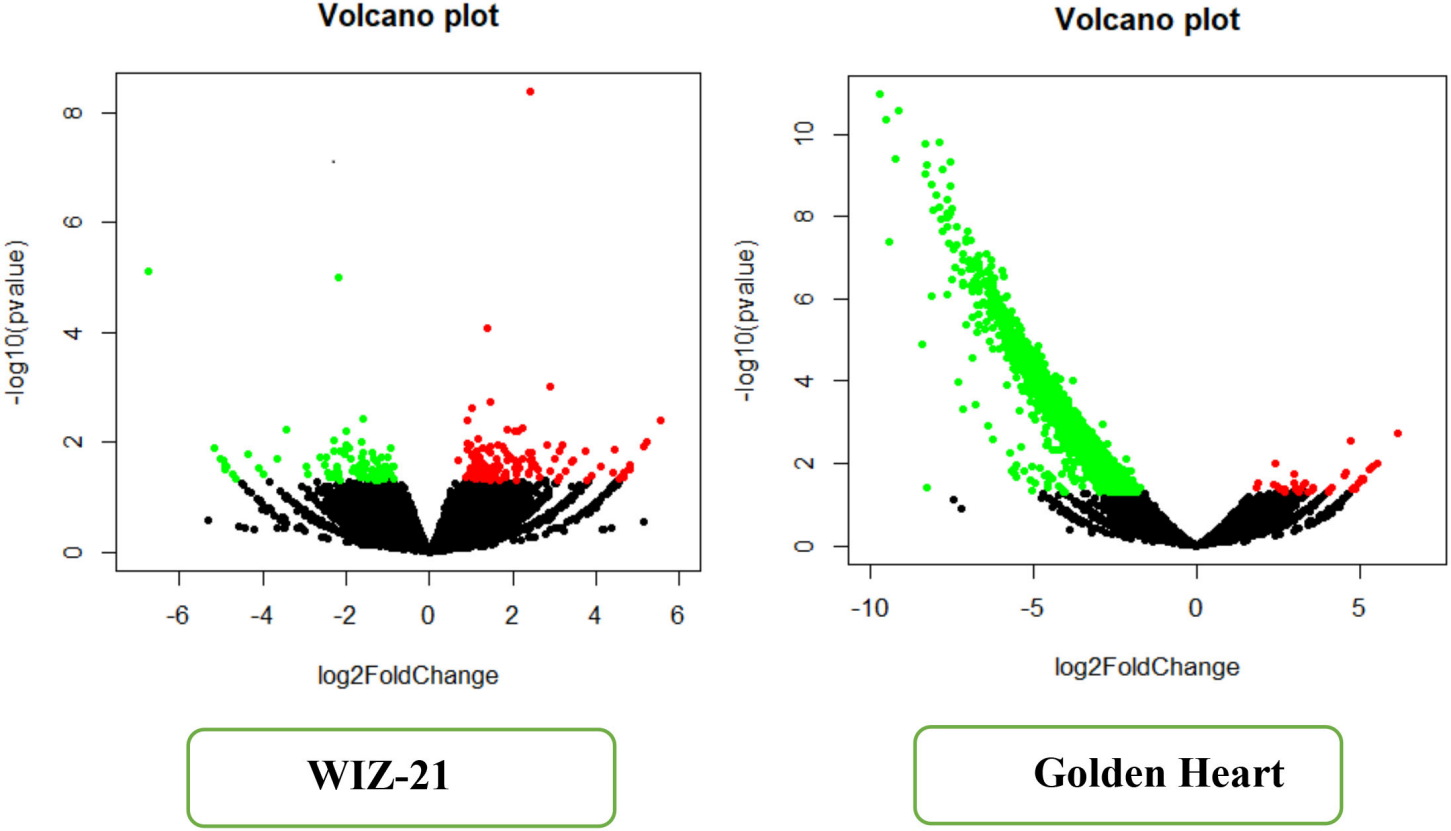
A volcano plot shows the DEGs for both cultivars under the foliar treatments SA and AA during Chilling stress.

In WiZ-21, the differentially expressed features are scattered in a plot that shows a symmetrical distribution around the zero log_2_ FC axis. The red color represents the upstream region of the gene cluster in the positive log_2_ fold-change area, while the green color indicates the downregulation of gene groups in the negative area. Remarkably, some genes exceed a -log_10_ p-value over 8, highlighting both statistically significant variation and substantial fold changes suggestive of a strong transcriptional response in WIZ-21. Unlike WiZ-21, the Golden Heart revealed an asymmetric distribution, where many dots were positioned in the negative log_2_ FC region, green color, reflecting that downregulation drives the transcriptional expression. The -log_10_ p-values were slightly above 10, focusing on highly significant DEG. Upstream genes displayed in red color, which were smaller in number and confined to small log_2_ FC values. This distribution suggests that the plant treated with SA+AA may largely suppress gene expression in Golden Heart; conversely, WiZ-21 exhibits a more balanced up- and downregulation of genes. The volcano plots present distinct transcriptional variation between varieties: WiZ-21 demonstrates both up- and downstream genes, although Golden Heart mainly expressed downregulation. Such differences may reflect physiological or molecular changes associated with genotype-specific regulatory pathways (**Figure 7**).

### 3.5 Gene expression profiling through hierarchical clustering analysis

Characterizations of DEGs subjected to Chilling stress. Differentially expressed genes across biological samples were characterized by FC standards based on the expression of assembled records. We identified differentially expressed genes as those exhibiting an absolute log_2_ FC >1 and (FDR)-adjusted p-value less than 0.05. The heatmap of Golden Heart (Treatment vs control), top 3923 DEGs, was visualized by the heatmap package in R Studio. While in Wiz-21, display the 210 Significant DEGs (Treatment vs Control). Golden Heart shows Maximum downregulation in treated plants, while the WIZ-21 interprets the high-rate Upstream genes in treated plants.

**Figure 8:**
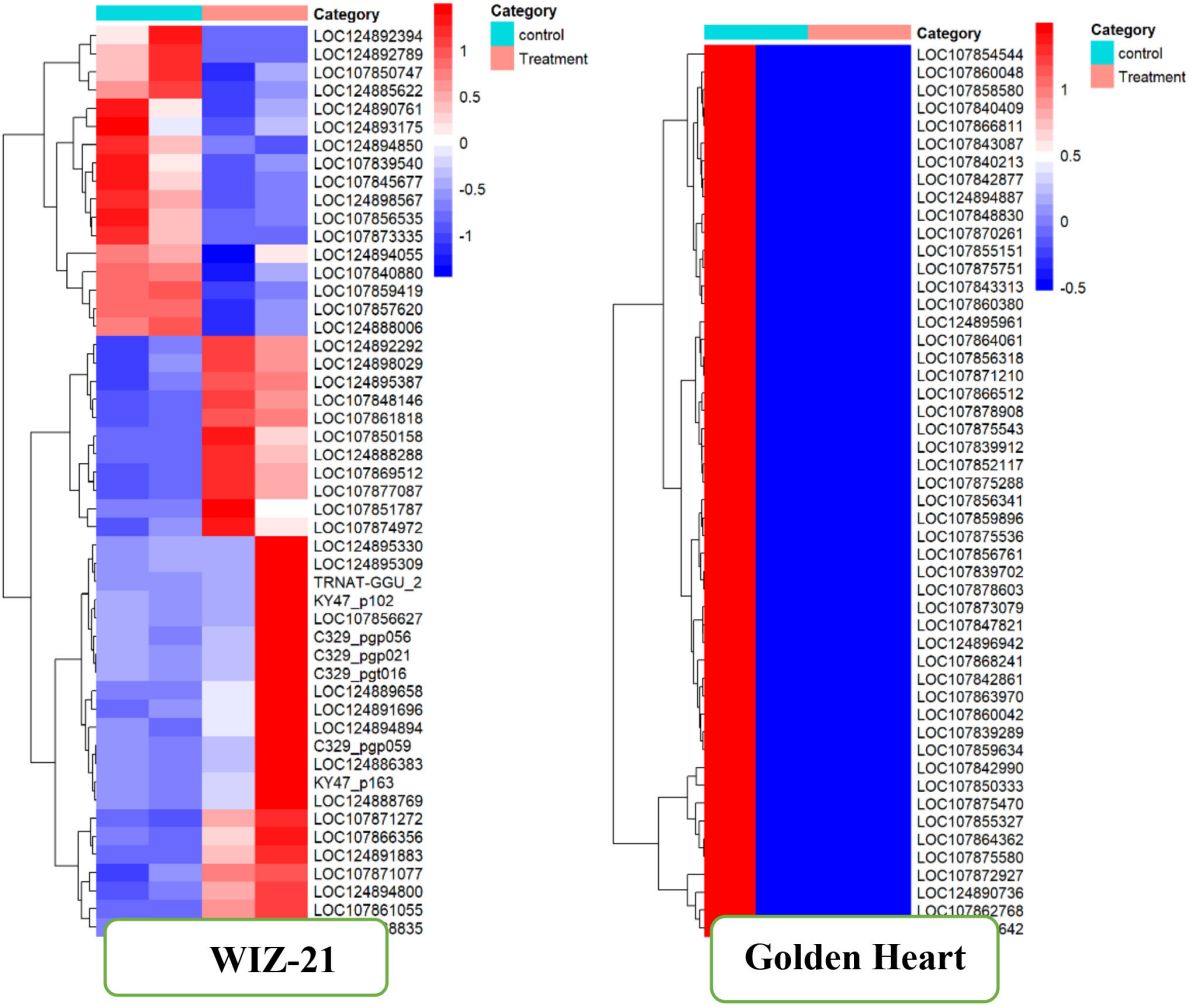
Effect of foliar treatment with control of DEEGs on both cultivars of Chilli under Chilling stress.

The analysis provides deep insight into the transcriptional remodeling stimulated by the treatment. A hierarchical clustering heatmap was produced to visualize the expression trend of differentially expressed genes (DEGs) contrasting the control and treated samples (**Figure 8**). Each gene is represented as a horizontal feature in the heatmap, while the color scale from blue to red indicates relative transcript abundance, in which blue shades reflect downregulation and red shades reflect upregulation. Before clustering, the expression data were normalized to allow consistent comparison across all samples.

In the Golden heart variety, these same genes exhibited a noticeable reduction in transcript abundance under treatment conditions, as reflected by the uniform blue shading. This specific color inversion suggests a broad suppression of transcriptional activity across the analyzed gene set in response to the applied treatment. The uniform pattern of downregulation indicated that the treatment induced a strong inhibitory effect on gene transcription, likely associated with the suppression of important physiological and metabolic processes. The magnitude and uniformity of this transcriptional response further suggest that genes are co-regulated and may be governed by a common upstream regulatory pathway that regulates gene expression patterns induced by environmental or stress-related factors (**Figure 8**).

#### 3.5.1 Cluster-specific expression and biological implications

The hierarchical clustering analysis demonstrated a distinct partitioning among the control and treatment groups, indicating that the implemented treatment provoked significant transcriptional modulation. Under untreated conditions, numerous genes, for instance, LOC124892934, LOC124892789, and LOC107850747, showed significantly high expression levels, by elevated red coloration. However, these genes were markedly downregulated (blue shading) in the treatment group, indicating that their transcription was significantly suppressed in response to the experimental condition.

Conversely, another set of genes, such as LOC124889156, LOC124894594, and C329_pg059, presented a comparatively low level of expression under control conditions but were significantly upregulated in response to the treatment. This contrasting expression pattern suggests that these genes are responsive to the applied treatment and may play important roles in physiological adjustments associated with adaptation and defense. The recorded transcriptional pattern key finds a bidirectional regulatory pattern, a broad change in expression, downregulation of genes expressed independently of external stimuli, accompanied by selective activation of stress-related transcripts.

The topmost cluster in the heatmap primarily comprises genes that exhibited high expression levels under control conditions but were markedly downregulated following treatment. Notable genes identified in this group, including LOC124892934, LOC124885637, and LOC107857333, are likely implicated in essential metabolic and housekeeping processes. Their downregulation under treatment suggests a strategic shift in metabolic priorities, whereby the plant limits energy-intensive growth processes to enhance defense and stress adaptation responses. Such transcriptional reallocation has been widely documented in plants experiencing abiotic stress conditions.

The Clusters located in the middle and lower regions contained genes such as KY47_p102, LOC124889156, and LOC124868363, which showed significant upregulation under the treatment condition in the WIZ-21 variety. These genes may be responsible for encoding proteins related to stress tolerance, such as transcriptional regulators, molecular chaperones, or enzymes involved in secondary metabolism. The co-expression pattern is particularly noteworthy of HSP-like (heat shock protein homologs, regulatory proteins (e.g., C329_pg021) and tRNA-related genes (e.g., TRNAT-GGU_2), which highlight the potential coordination by collective transcriptional regulators, potentially associated with stress-related signaling pathways such as heat shock factors (HSFs) or WRKY transcription factors.

The hierarchical dendrogram of golden heart reveals a compact clustering of genes with similar expression profiles, underscoring a strong co-expression relationship among these transcripts. Notably, genes such as *LOC107854544*, *LOC107860048*, *LOC107854580*, *LOC107863611*, and *LOC124894887* exhibit upregulation under control conditions and significant downregulation under treatment. These transcripts likely constitute integral components of fundamental metabolic, structural, or regulatory pathways that are downstream to conserve cellular energy or redirect resources to support stress adaptation processes.

The transcriptional repression of genes such as *LOC107865211*, *LOC107875628*, and *LOC107854153*, which are putatively involved in transcriptional regulation, protein synthesis, or photosynthesis metabolism, reflects a widespread decrease in cellular biosynthetic activity. This response is characteristic of plant adaptive response to abiotic stress, wherein growth-associated pathways are temporarily downstream, although protective mechanisms are activated at alternative molecular levels, such as post-transcriptional or post-translational regulation.

The consistent downregulation observed across all clusters suggests that the treatment may have triggered a state of transcriptional activity, characterized by a global suppression of gene expression. This phenomenon is commonly associated with plant responses to abiotic stress like cold, salinity, or drought, wherein metabolic activity is suppressed to optimize energy utilization and preserve essential fundamental physiological functions.

### 3.6 Function annotation of DEGs Induced by SA+AA under chilling stress in ornamental Chillies

**Figure 9:**
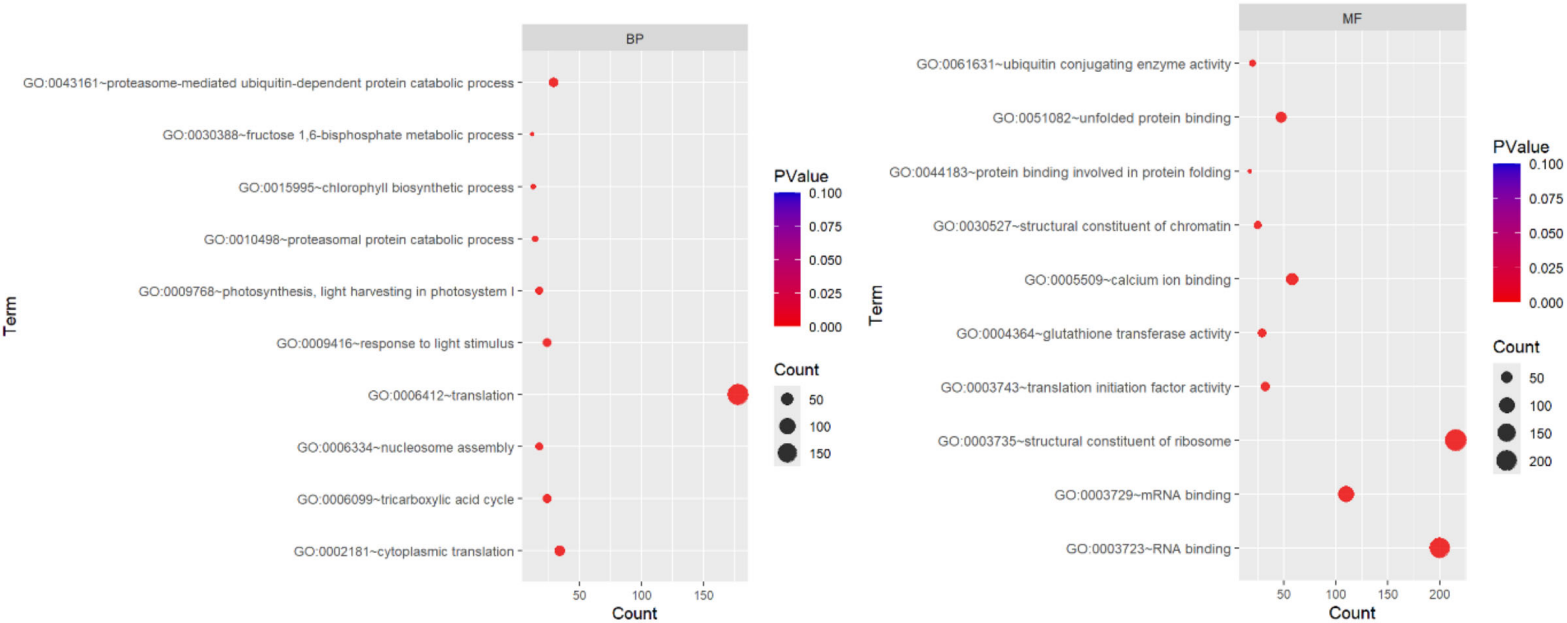
Biological process and Molecular function analysis of DEGs between control and treatment under Chilling stress in Golden heart.

**Figure 10:**
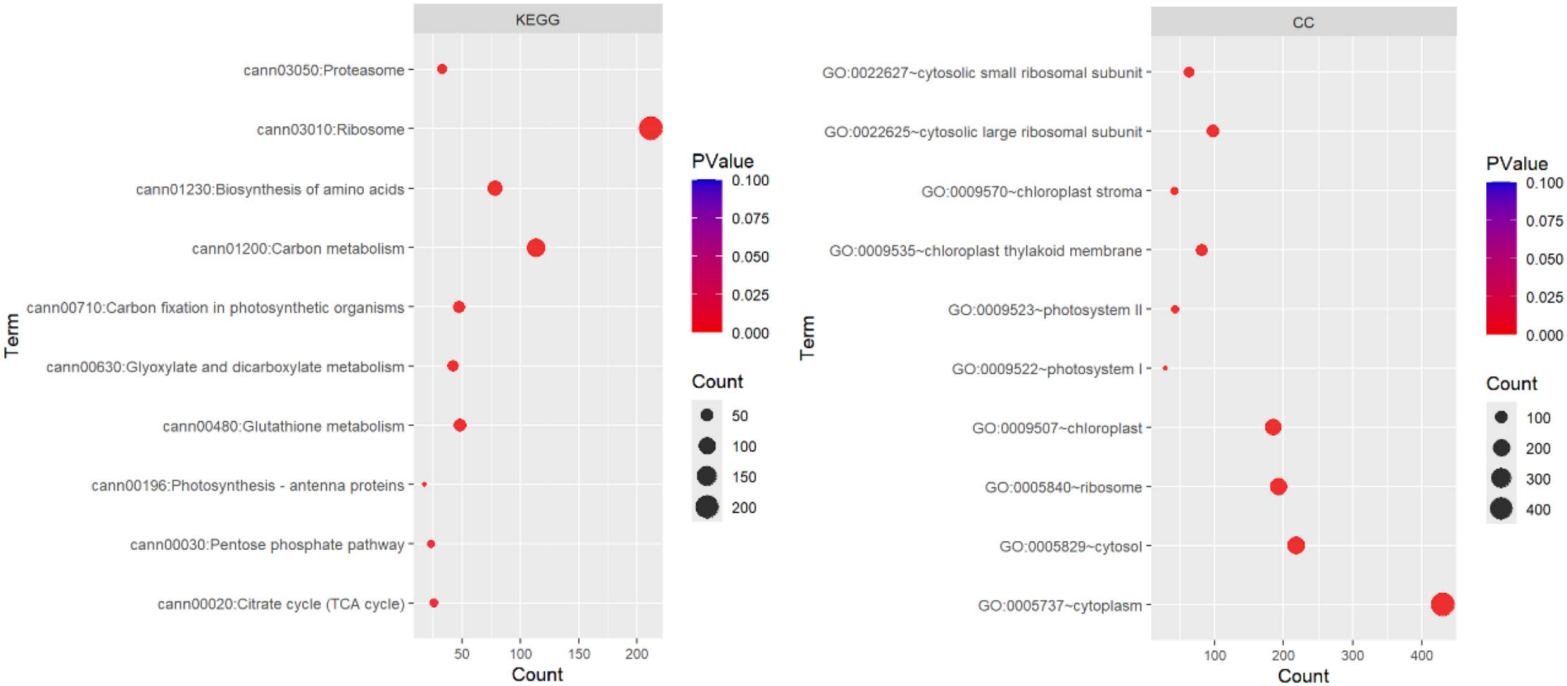
KEGG pathway and Cellular component classification of DEGs between control and treatment under Chilling stress in Golden heart.

**Figure 11:**
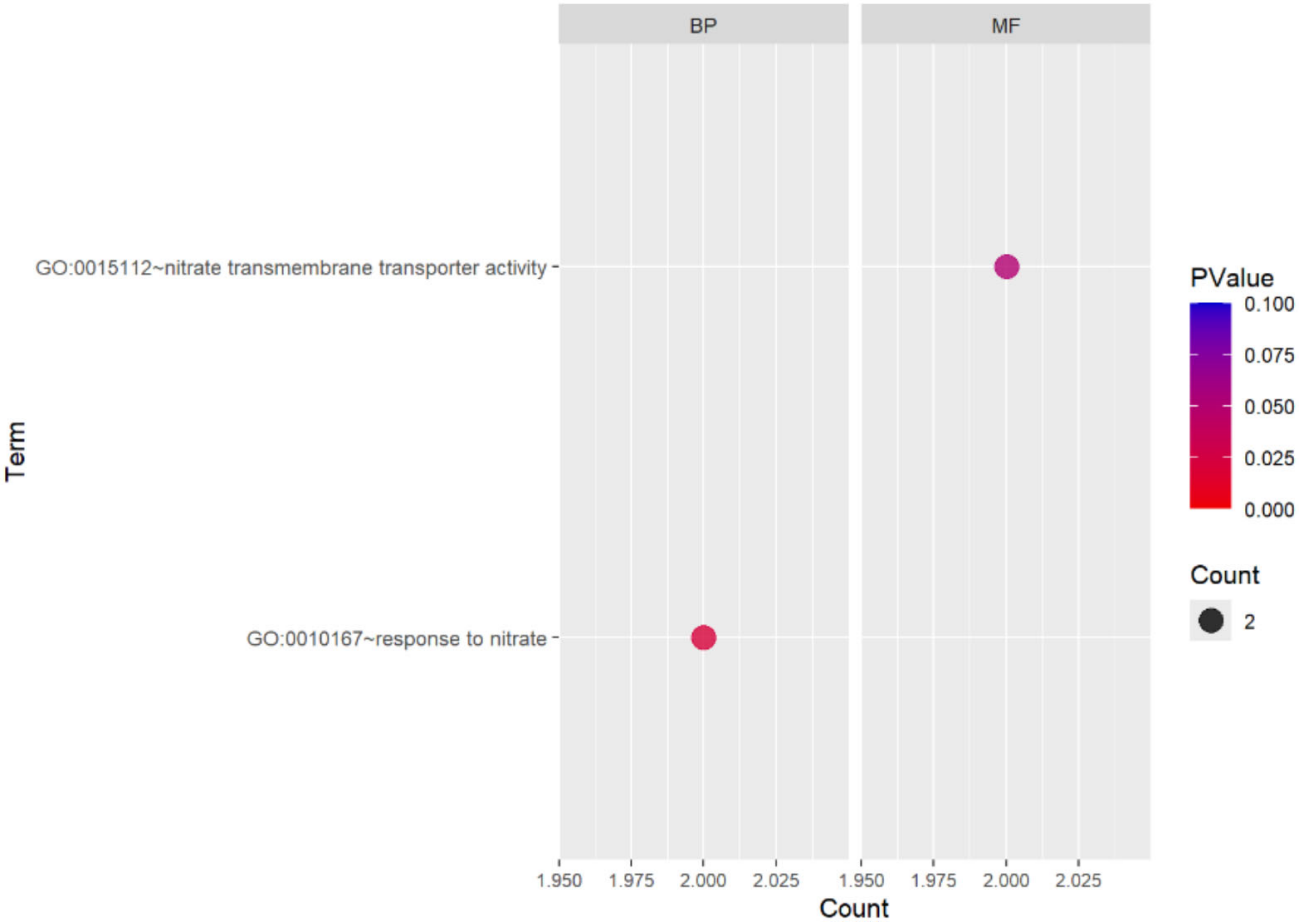
Biological process and Molecular function analysis of DEGs between control and treatment under Chilling stress in WIZ-21.

Gene Ontology (GO) functional analysis of DEGs revealed significant enrichment of terms associated with defined cellular components. The major enriched components, such as the cell wall, extracellular region, and plasma membrane, indicate that a substantial fraction of the regulated genes were site-specific to or involved in structural and membrane-associated components. We examined the functional profiling of DEGs using gene ontology enrichment analysis, which demonstrated the biological process (BP), cellular component (CC), and molecular function (MF) groups for the 3923 DEGs of Golden Heart. Among these, 1791 genes were significantly enriched in molecular function, 893 genes were significantly enriched in KEGG pathway, and 1736 genes were significantly enriched in cellular components. Furthermore, 1242 genes were significantly involved in biological processes. At the cellular process, the terms that were substantial enriched cytoplasm (GO: 0005737; 431 genes), cytosol (GO: 0005829; 218 genes), ribosome (GO: 0005840; 192 genes), chloroplast (GO: 0009507;185 genes), chloroplast thylakoid membrane (GO: 0009535; 85 genes), and photosystem II (GO: 0009523; 42 genes). Inside the molecular function domain, the term that was notably enriched in RNA binding groups (GO: 0003723; 200 genes), Structural constituents of ribosome (GO:0003735; 216), calcium ion binding (GO: 0005509;58), and glutathione transferase activity (GO: 0004364) with 29 genes. In the context of the biological process category, highly enriched terms comprised translation (GO: 0006412; 178 genes), chlorophyll biosynthetic process (GO:0015995), and response to light stimulus (GO: 0009416) with 13 and 24 genes, respectively (**Figure 9**) Analysis of DGEs between the control and treatment groups revealed10 pathways, with a KEGG pathway annotation, were significantly altered: Citrate cycle, pentose phosphate pathway, glutathione metabolism carbon metabolism, photosynthesis-antenna proteins, glyoxylate and dicarboxylate metabolism, Pyruvate metabolism, carbon fixation in photosynthetic organism, biosynthesis of amino acids, ribosome, proteasome, Cysteine and methionine metabolism, porphyrin metabolism, Glycolysis/Gluconeogenesis metabolism, carbon fixation in photosynthetic organisms, ubiquitin mediated proteolysis pathway (p < 0.05; **Tables 2**, **3**; **Figure 10**). We analyzed the gene expression of DEGs using functional annotation analysis, which presents the BP and MF annotations for the 210 DEGs of WIZ-21. Out of these, 16 genes were highly enriched in molecular function, and 4 genes were prominently enriched in BP. In molecular function, the terms that were enriched considerably were nitrate transmembrane transporter activity (GO: 001512; 2 genes). Inside the biological process category, the annotation was notably enriched in response to nitrate (GO: 0010167), including 2 genes (**Figure 11**).

**Table 2:** Different expressed genes (DEGs) annotation function of golden heart (V2) in control and treated.

Locus ID	Gene ID	Name	Log 2-FOLD change	Regulation	Functions
LOC124889831	PHT68370	Impaired in glycan perception 4	4.970169521	Up	Defense response by callose deposition
LOC124893127	PHT77936	COBRA-like protein 6	4.955951032	Up	Plant-type cell wall cellulose biosynthetic process
LOC124899618	PHT88862	Cysteine-Rich rlk (Receptor-like Protein kinase) 8	4.55570289	Up	Defense response to the bacterium
LOC124885318	PHT77751	DUF247-1	3.440663503	Up	Cell wall polysaccharide metabolic process
LOC107875206	PHT75156	Rho-GTPase activating protein 2	-1.71570979	Down	Signal transduction
LOC107850774	PHT65230	DUF668 domain-containing protein	-3.07003711	Down	Positive regulation of growth
LOC107845910	PHT62890	B-BOX Domain Protein 29	-3.30005273	Down	Response to cold
LOC124894055	PHT84770	ACTIN-12	1.83180401	Up	Cytoskeleton organization
LOC124892188	PHT81066	Nuclear Factor Y (NF-YB10),	2.387200526	Up	Regulation of transcription by RNA polymerase II
LOC107853651	PHT82380	ankyrin repeat-containing protein ITN1-like	2.560580655	Up	Response to abiotic stress
LOC124892604	PHT72950	Uncharacterized protein	2.716338514	Up	Signal transduction
LOC107841439	PHT83609	MAD4	2.730999058	Up	Sterol biosynthetic process
LOC107861424	PHT57513	TUB5, Tubulin Beta-5 Chain	3.002376259	Up	Microtubule-based process
LOC107866247	PHT81066	Proline Iminopeptidase (PIP)	3.129644742	Up	Response to Abiotic Stress
LOC124891918	PHT86602	Adp-Ribosylation factor b1b, (ARFB1B)	3.194993684	Up	N-terminal protein myristoylation
LOC124885318	PHT71473	Lipid Phosphate Phosphatase 3, LPP3	3.440663503	Up	Phospholipid metabolic process
LOC124897351	PHT72461	MYB 2,	4.842497949	Up	Regulation of DNA-templated transcription
LOC124894760	PHT74827	Rhodanese-like protein, STR10	4.795507023	Up	Response to cold
LOC124889907	PHT72461	HOMEOBOX 7 (HB-7)	4.858078214	Up	Abscisic acid-activated signaling pathway
LOC107846163	PHT91746	ATPDS5D, PDS5D	4.603946571	Up	DNA recombination

**Table 3:** Different expressed genes (DEGs) annotation function of WIZ-21 (V1) in control and treated.

Locus ID	Gene ID	Name	Log 2-FOLD change	Regulation	Functions
TRNATGGU_2	PHT73025	Sucrose-6f-phosphate phosphohydrolase 2 (SPP2)	2.894033841	Up	Sucrose biosynthetic process
C329_pgp059	PHT63337	Acetyl-coenzyme A carboxylase carboxyl transferase subunit beta (AccD)	0.910619945	Up	Fatty acid biosynthetic process
C329_pgp021	PHT61618	NAD (P H-quinone oxidoreductase subunit 5 (NDHF)	1.17089115	Up	ATP synthesis coupled electron transport,
KY47_p163	PHT74813	Brassinosteroid-signaling kinase 1 (BSK1)	2.815083217	Up	Brassinosteroid mediated signaling pathway
C329_pgp056	PHT78704	Potassium/proton antiporter CemA, (CemA) YCF10	1.872825794	Up	CemA (Potassium/proton antiporter CemA), YCF10
KY47_p006	PHT61765	Small auxin Upstream rna 79 (SAUR79)	2.06828001	Up	Retrograde vesicle-mediated transport,
KY47_p054	PHT78705	Transmembrane amino acid transporter family protein	2.886843892	Up	Amino acid transmembrane transport
LOC124897622	PHT79977	Uncharcterized protein	-2.14881920	Down	Carbohydrate metabolic process
LOC124897283	PHT79049	Adenyl succinate synthase (ADSS)	3.834690564	Up	AMP biosynthetic process
LOC124891566	PHT79049	3-Keto-acyl-coenzyme A thiolase 5 (KAT5)	-1.30987640	Down	Fatty acid oxidation
LOC107875182	PHT24484	Glucuronosyltransferase-like 4 (GATL4)	3.095153218	Up	Pectin biosynthetic process
LOC107857836	PHT89425	Light stress-regulated 1 (LSR1)	4.68036308	Up	Response to high light intensity
LOC107847085	PHT67869	Neutral Ceramidase 2 (ATNCER2)	2.647600838	Up	Ceramide catabolic process
LOC124898072	PHT75066	Cysteine-rich rlk receptor-like protein kinase 8 (CRK8)	3.131163131	Up	Response to defense, protein phosphorylation
LOC107870318	PHT87990	Responsive to desiccation 22 (RD22)	4.40726473	Up	Response to abscisic acid, Response to abiotic stress
LOC107873335	PHT64454	BCE2, Dark inducible 3 (DIN3), LTA1	-1.94399203	Down	Response to sucrose
LOC107859419	PHT61751	Non-host resistance 2b (ATNHR2B)	-2.29914774	Down	Defense response to the bacterium
LOC107866356	PHT61330	Zinc finger protein 1, (ZP1)	3.10800785	Up	Regulation of DNA-templated transcription,
LOC107868835	PHT80431	Uncharacterized protein	2.46812203	Up	Mevalonate pathway
LOC124891696	PHT83812	Nuclear Factor Y, (NF-YB10	2.403503201	Up	Regulation of transcription by RNA polymerase II,
LOC107864019	PHT86196	REF/SRPP, LD-associated protein 3, (LDAP3)	1.655545331	Up	Positive regulation of response to water deprivation
LOC124894292	PHT54623	Rhodanese-like protein (STR10)	-4.90269701	Down	Response to cold
LOC124897948	PHT83324	Ribosomal protein S11 (US11C), Ribosomal protein (RPS11)	4.804146849	Up	Involved in the maturation of SSU-RNA from the tricistronic rRNA transcript
LOC107851517	PHT61814	Ethylene-responsive transcription factor (ERF027)	2.593927677	Up	Glucosinolate metabolic process,
LOC124886210	PHT96131	Embryo Defective 2734 (EMB2734)	-3.99241368	Down	Embryo development ending in seed dormancy

### 3.7 The qRT-PCR validation of candidate genes

**Figure 12:**
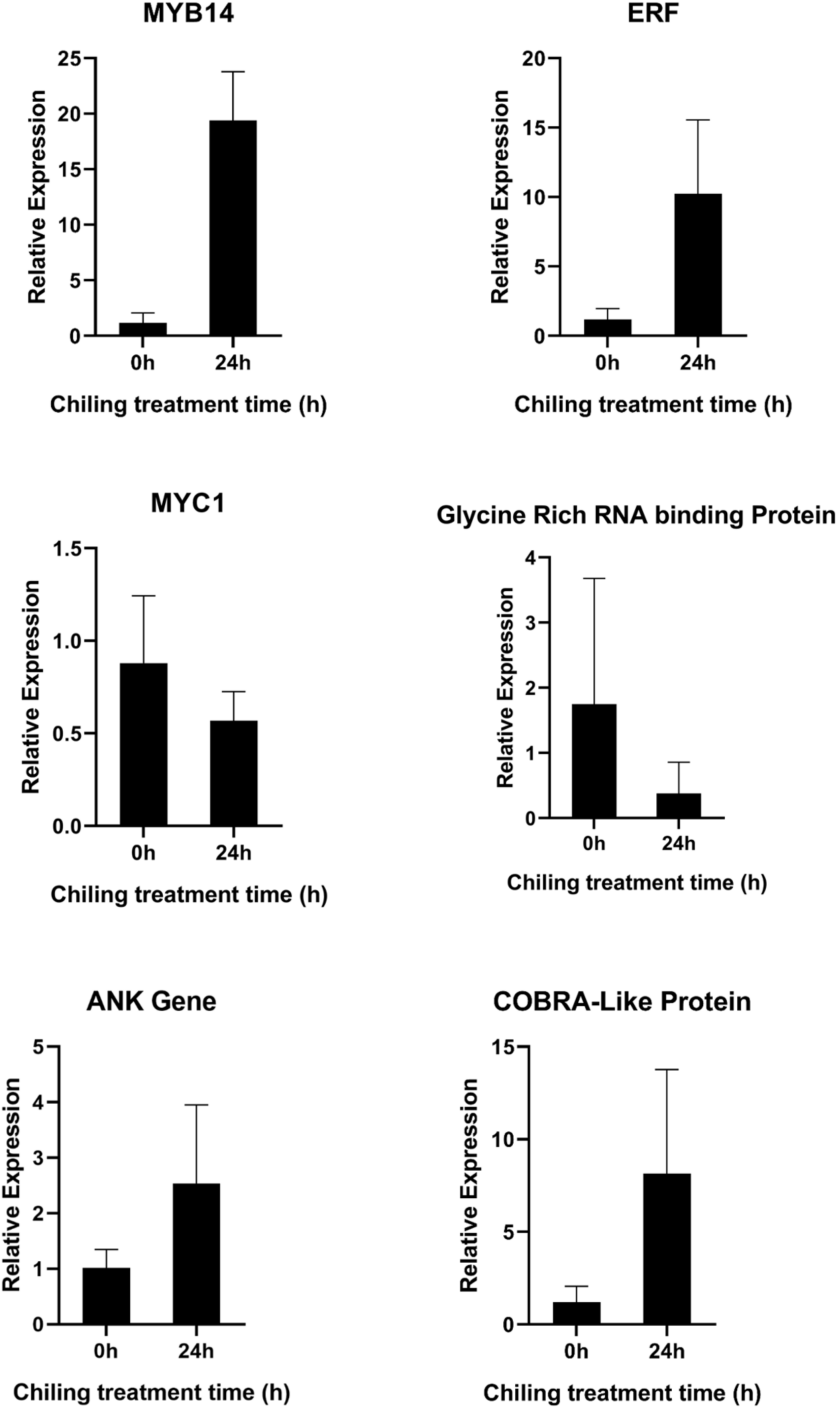
Quantitative real-time PCR (qRT-PCR) analysis of six selected DEGs from both WIZ-21 and Golden Heart cultivars related to SA and AA treatment under Chilling stress. The bar graphs represent the expression ratios of the genes before and after foliar treatment under Chilling stress. Statistical significance is indicated at *p* ≤ 0.05 and *p* ≤ 0.01.

From empirical data validating the expression of DEGs identified through RNA-sequencing, we screened six key genes involved in the ethylene and ABA signaling pathway, Cellulose biosynthesis, and post-transcriptional gene (ERF, COBRA-like protein, glycine-rich RNA binding protein) and Ras/MEK/ERK (MAPK), and PI3K/Akt/mTOR pathways, ABA signaling pathway (MYC, MYB14, and ITN1). Validation by qRT-PCR illustrated that most candidate genes associated with ABA signaling, ethylene pathway, and cellulose biosynthesis were strongly expressed in treated leaves with SA+AA as compared with the control unit; few genes were expressed in downregulation with the treatment of SA+AA in comparison to control ornamental chilli plants Serendipitously, the gene expression pattern by the qRT-PCR analysis were consistent to the FPKM in RNA -seq analysis (**Figure 12**).

## 4 Discussion

Chilling stress is a significant abiotic factor that inhibits plant growth and development. The accurate regulation of internal phytohormone by the exogenous application of plant growth regulators has emerged as an effective agronomic strategy to increase resilience under chilling stress (Waititu et al., 2021). Chilling stress induces metabolic and physiological disturbance in plants, characterized by a significant decline in photosynthetic performance under low temperature stress. The adverse effects increase as the temperature decreases, and the stress period is prolonged (Jiang et al., 2020). Exposure to Chilling stress negatively influences photosynthetic activity, resulting in stunted root growth and insufficient mineral supply (Xu et al., 2023). Waleed et al. (2025) and Yousif et al. (2026) demonstrate that drought conditions significantly alter plant morphology, including reduced plant height, leaf area, and biomass, while promoting adaptive traits such as increased root length and root-to-shoot ratio. These changes enhance water uptake efficiency and help plants survive under limited moisture availability. Osmotic imbalance caused by membrane lipid phase transitions can impede cytoplasmic flow and increase electrolyte leakage, disrupting the transmembrane ion gradient and altering the conformation of key enzymes, culminating in reduced catalytic activity (Lu et al., 2023). Plant hormones serve as key regulators of growth and development. When subjected to low-temperature stress, endogenous hormone levels are modulated, which, in turn, affects the adaptive capacity of plants under low-temperature conditions (Zhang et al., 2022). Previous research has demonstrated that foliar application of SA plays a pivotal role in improving plant physiology and tolerance to abiotic stress (Li and Wang, 2021; Decsi et al., 2025). Previous studies indicate that salicylic acid (SA) may act as a potent signaling compound that promotes systemic resistance by regulating defense mechanisms and strengthening antioxidant responses. Salicylic-acid–derived treatments emerge as environmentally friendly approaches for alleviating biotic stress in forage grasses while enhancing crop resilience (Guamán Rivera et al., 2026). Ascorbic acid (AA) performs two distinct functions by acting as a direct antioxidant and serving as a cofactor in numerous enzymatic reactions, synergistically enhancing a plant’s ability to withstand oxidative stress (Hasanuzzaman et al., 2019). Excessive accumulation of ROS can induce cellular and tissue damage and markedly influence transcriptional regulation under low temperature stress (Dreyer and Dietz, 2018; Zhang et al., 2022). Reactive oxygen species are metabolic byproducts and participate in different signaling pathways in organisms, with hydrogen peroxide (H2O2), superoxide anion Radical O2· and hydroxyl (-OH). Plants may produce ROS in the metabolic process in abiotic stress (Miller et al., 2010).

Evidence suggests that external application of hormones SA and AA can increase the functions of antioxidant enzymes such as CAT and SOD to mitigate the harmful effects of abiotic stress by decreasing ROS-driven lipid peroxidation (MDA), thereby enhancing the plant’s tolerance to Chilling stress. Studies have shown that SA and AA stimulate the upregulation of stress-responsive genes of these antioxidant enzymes in plants’ response to abiotic stress, leading to a decline in ROS accumulation and reduced lipid peroxidation levels (Ortega et al., 2024; Noreen et al., 2021). Ghani et al. (2020) reported that foliar application of SA enhances canola biomass and mitigates the adverse effects of abiotic stress. Exogenous application of SA increased photosynthetic pigments and reduced ROS activity under adverse environmental conditions in tomato (Jahan et al., 2019). Our results align with those of Jalili et al. (2023), who found that the use of SA can improve the cold resistance of several grape varieties by fortifying the antioxidant defense system of plant cells, maintaining cell membrane integrity under low temperature stress.

The abscisic acid (ABA) signaling system has been characterized as a key regulator of plants exposed to abiotic stress, accompanied by significant alterations in gene expression and physiological adaptations. Low temperature stress stimulated abscisic acid (ABA) biosynthesis in tomato plants and enhanced the concentration of endogenous ABA. Remarkably, ascorbic acid (AA) treatment might enhance the accumulation of ABA within the plant under low temperature stress. The effect of externally applied AA on endogenous ABA abundance is likely contributed to a potential interaction between AA and phytohormones (Ye et al., 2012). Ascorbic acid foliar spray promotes cold tolerance in tomatoes with respect to hormonal contents, through crosstalk between AA and phytohormones that regulate ROS and subsequent developmental adaptation in plants (Wang et al., 2024). Candidate gene analysis suggested the involvement of genes related to hormone signaling, membrane stability, antioxidant defense, and stress-responsive transcription factors (Pan et al., 2023).

Chilling stress provokes significant remodeling of the plant cell wall, a mechanism through which COBRA-LIKE (COBL) proteins act as key regulators of cellulose microfibril deposition and crystallinity. Genome- and transcriptome-wide studies have consistently observed that COBL family proteins exhibit cold-induced expression across a range of plant species, correlating with cell wall structural reconfiguration events pivotal for cold acclimation (Sajjad et al., 2023; Qiu et al., 2023).

COBRA-family protein is distinguished by three structurally preserved regions, including a N-terminal signal peptide involved in secretion, a Cysteine-rich CCVS domain, and a hydrophobic C-terminal domain responsible for GPI anchoring (Deng et al., 2025). TaCOBL6A2 exhibits all these motifs and shows significant sequence resemblance and conserved transcript profile to its homologs. Furthermore, the TaCOBL6A2 protein was strongly regulated in the early stages of grain formation and development. Key cis-regularity segments, such as MYB-binding site, DRE core (DREB-binding site), and STRE (Stress-Responsive Element) have been documented to be involved in the regulation of plant stress responses (Niu et al., 2020; Deng et al., 2020; Adak et al., 2023). The presence of these conserved elements in TaCOBL6A2 and its homologs may support a putative role in co-regulated responses to environmental stress, although it presents some changes in the expression profile under distinct stress conditions. Prior investigations have shown that TaCOBL6A2 participates in adaptive responses to diverse stress conditions, including heat, salt, drought, and Chilling stress. This capacity to respond to multiple abiotic stresses aligns with earlier findings that specific COBL family proteins improve abiotic stress resilience by cellulose accumulation and modifying the cell wall (Li et al., 2022; Sun et al., 2022). Our observations indicate that exogenous combined spray of SA and AA have been shown to boost the expression of COBRA-LIKE (COBL) protein under Chilling stress. Salicylic acid (SA) potentially regulates stress signaling pathways, although ascorbic acid (AA) increases antioxidant defense activity and promotes cell wall remodeling. This upregulation contributes to anatomical adaptations critical for cold tolerance in ornamental Chilli plants.

Ethylene response factors (ERFs) are transcription factors unique to plants and members of the AP2/ERF superfamily, which are critical in stress responses through regulation of downstream target genes (Zhao et al., 2018). Overexpression of AtERF102 to AtERF105 in Arabidopsis enhances cold resistance in transgenic lines by activating the CBF pathway. We have also observed upregulation of ERF genes in our study, which supports the previous statement by Illgen et al. (2020). The AP2/ERF transcription factors are vital for plant stress adaptation, and the DREB subfamily contributes to abiotic stresses by regulating the expression of cold-, drought-, and salt-responsive genes (Feng et al., 2020). In the Arabidopsis DREB-A1 category of genes, CBF1/CBF2/CBF3 function as principal regulators of low temperature stress response; their elevated expression notably upregulates the COR (cold-responsive) genes and improves resistance to cold (Qian et al., 2024; Rosenkranz et al., 2025**;**Lee et al., 2024). Similarly, SlERF2 in tomato is suggested to improve tolerance to chilling stress by promoting ABA biosynthesis and activating the CBF-dependent signaling pathway (Yu et al., 2023). The MYB transcription factor (TF) family represents one of the most evolutionarily distributed groups in the plant kingdom. In Arabidopsis thaliana and rice, more than one hundred MYB members have been investigated, which specifically participate in Chilling stress adaptation.

These Transcription Factors are characterized into four distinct subgroups as inferred from the structure of the DNA-binding motif, encompassing 1R-MYB, 4R-MYB, R2R3-MYB, and R1R2R3-MYB (Su et al., 2014). Several Members of the MYB protein are associated with a broad spectrum of physiological and developmental processes, such as cell cycle regulation, flower and seed formation, metabolic regulation, hormone-dependent signaling, and stress tolerance (Li et al., 2015). Accumulating evidence has demonstrated that the MYB gene expression induced in response to low temperature stress is mediated through the CBF/DREB pathway (Mehrotra et al., 2020).

Additionally, recent research has demonstrated that R2R3 MYB TFs can downregulate the expression of COR genes by activating the expression of CBFs, a core transcription factor involved in Chilling stress, establishing an intricate regulatory network to promote their regulatory potential (Wang et al., 2021a, 2021b). MdMYB23 improves cold tolerance in apple calli through direct interaction with the promoters of MdCBF1 and MdCBF2, thereby inducing their expression. Likewise, overexpression of MdMYB73 in apple calli enhanced the expression of MdCBF3, MdCBF4, and MdCBF5, suggesting that MdMYB73 promotes cold resistance through the cold-responsive CBF pathway. Besides, MdMYB124 and MdMYB88 target MdCCA1 and MdCSP3, respectively, upregulating genes related to cold tolerance and Chilling stress response in apple (An et al., 2020; Zhou et al., 2024). In our study, the upregulation of the MYB gene was observed, which may play a complementary or regulatory role in the chilling stress response network in ornamental Chilli plants.

The CBF (CRT-binding factor)/DREB1 (DRE-binding protein) proteins are AP2-type TFs that interact with the core sequence CCGAC of CRT/DRE cis-regulatory elements to promote gene expression in response to drought and Chilling stress. As CBF/DREB1 functions upregulation in the Chilling stress signaling network, additional TFS are essential to regulate their activation in response to Chilling stress. Numerous factors that might regulate CBF/DREB expression have been molecularly identified in Arabidopsis (Ohta et al., 2018).

ICE1 mutants exhibit suppressed transcription of CBF3/DREB1A along with its downstream potential genes, which correlates with an enhanced sensitivity to both chilling and freezing stress. ICE1, a MYC-like basic helix–loop–helix (bHLH) transcription factor, links directly to canonical MYC cis-elements in the CBF3/DREB1A promoter, thereby regulating its expression. Through this regulatory mechanism, ICE1 serves as a critical upstream activator of the CBF-mediated cold response pathway, enabling the expression of downstream genes that contribute to cold tolerance in plants. In contrast, the expression of CBF1/DREB1B, instead of CBF3/DREB1A, is predominantly regulated by ICE2, a homolog of ICE1. Mehrotra et al. (2020) reported that ICE1 and ICE2 play a crucial role in the transcriptional regulation of CBF/DREBI genes under Chilling stress. In Arabidopsis, MYC67 and MYC70 interact with ICE1 and function as repressors of cold-signaling pathways. The ICE1-associated cold signaling mechanisms are retained in tomato and rice. Correspondingly, MYC co-regulatory proteins potentially stimulate cold resistance by activating the expression of CBF/DREB and cold-responsive genes across multiple species (Ohta et al., 2018; Liu et al., 2024). Our results correlate with a previous study. Abscisic acid (ABA) functions as a core regulator of plant responses to environmental stress, orchestrating and coordinating multiple physiological and developmental processes to enhance stress tolerance (Wani and Kumar, 2015). ABA-dependent and ABA-independent regulatory mechanisms likely mediate the expression of stress-responsive genes. Different TFs may contribute significantly to stress signal transduction, particularly AP2/ERF, WRKY, NAC/ZF-HD, and MYC/MYB (Hoang et al., 2017). ABA-dependent pathway facilitates cold acclimation by positive regulators such as the AREB/ABF bZIP, and MYC/MYB. ABA-independent signaling pathway suggests possible modulation of stress acclimation via the CBF/DREB, ERF, and the NAC and ZF-HD. However, some results are consistent with the presence of both ABA-dependent and ABA-independent pathways of stress response that contribute to the AP2/EREBP (ERF) proteins (Wani et al., 2025).

Glycine-rich RNA-binding proteins (GR-RBPs), defined by an N-terminal RNA-binding region and a C-terminal glycine-rich region, have been widely acknowledged as major regulators of post-transcriptional gene expression in plants, specifically triggered by abiotic stresses (Lewinski et al., 2024).

GRPs are classified into three major subgroups, I, II, and IV, specifically subclass IV, which are involved in the regulation of phytohormone-mediated signaling pathways, including salicylic acid, abscisic acid (ABA), and ethylene. Abscisic acid (ABA) acts as a central signaling molecule that integrates plant growth regulation with physiological and stress-responsive pathways. Consequently, AtGRP7 is implicated in mediating the ABA-dependent signaling, as well as in stress responses. Both AtGRP7 and AtGRP8 are involved in oxidative stress, and since ABA may trigger gene downregulation, a negative feedback loop may exist between ROS and ABA signaling pathways. Moreover, the Arabidopsis zinc finger containing GRPs (atRZ-1a) participates in ABA signaling and stress responses (Czolpinska and Rurek, 2018). Overexpression of MpGR-RBP1 in Arabidopsis reduced the level of ROS under salinity stress (Tan et al., 2014). In pepper, CaGR-RBP1 downregulates the CaPIK1-mediated cellular death and defense signaling by the suppression of ROS production (Kim et al., 2015).

Chilling stress influences CAT and mitochondria-encoded SOD through AtGR-RBP2. In Arabidopsis, CsGR-RBP3 overexpression attenuates ROS levels and enhances SOD expression and antioxidant enzyme activities relative to wild-type plants. This indicates that elevated antioxidant response in transgenic Arabidopsis has partial effects on strengthening resistance to chilling stress. Although the upstream CAT and SOD, two primarily ROS-scavenging enzymes, supported chilling tolerance in transgenic Arabidopsis, the overall response is facilitated by a network of interconnected defense pathways. To investigate the impact on other defense pathways that are modulated, we examined the expression of nine Arabidopsis genes—AtCOR47, AtCOR15b, AtPR1, AtHSP20, AtCML30, AtRD29A, AtNIA2, AtRH9, and AtPHR1—in transgenic Arabidopsis lines overexpressing cucumber CsGR-RBP3. Collectively, these genes participate in cold acclimation, systemic acquired resistance, and the response to ROS, and play key roles in RNA biogenesis and the DNA repair pathway (Wang B et al., 2018). Ankyrin-repeat proteins (ANKs) represent a major protein superfamily in plants and are essential regulators of responses to environmental and pathogenic stress. The ANK gene ITN1 might stimulate ABA-dependent ROS production and increase abiotic stress tolerance (Hou et al., 2025).

In the PmvANKs and PmANKs, hormone-responsive promoter elements are widely distributed in gene promoters, indicating a role in hormone-responsive gene expression. The promoter regions of the ANK gene are enriched with elements involved in low temperature and drought stress. Homologous hormone and stress-inducible elements, including those that are likely to be dependent on auxin, ABA, and drought stress, have been found in Chillies (Lopez-Ortiz et al., 2020).

The regulatory domains of PmvANK43 and PmvANK46 both comprised cis-regularity motifs that are hormone-sensitive, for instance, JA and ABA, together with elements linked to the cell cycle regulation (Miao et al., 2025). Prior research has shown PmANKs that contribute to salicylic acid metabolism, including NRP1 regulation. In citrus sinensis, ANK family members boost salicylic acid accumulation (Li, 2019). In Arabidopsis, the interaction between ANK-IQ proteins and CBF genes regulates cold tolerance by reducing SA levels (Kim et al., 2017).

ANK repeat–receptor-like proteins are involved in plant immune regulation; however, AKR2 plays a major role in ROS detoxification and metabolism. The OXIDATIVE STRESS 2 protein may further serve as an activator in a stress-related signaling pathway (Lopez-Ortiz et al., 2020). Chilling stress stimulates the MDA accumulation, thereby affecting the membrane stability (Wang Z et al., 2018; Santini et al., 2013). Wang (2021) declared that an elevated MDA level is usually inversely linked with cold tolerance. In cotton, ANK proteins act as a regulator in plastid differentiation and cell differentiation, as well as morphogenesis and organogenesis processes during plant growth (Hu et al., 2019). This regulation is achieved by prompting the ethylene, auxin, and anthocyanins biosynthesis pathway. Furthermore, ANK proteins are an integral part of stress adaptation pathways. Yan et al. (2002) discussed that Arabidopsis ANK proteins participate in the oxidative stress defense system by facilitating H2O2 scavenging and enhancing plant abiotic stress tolerance through ABA-mediated regulation of ROS homeostasis. In Glycine max, the ankyrin-type proteins trigger genes related to environmental stresses such as salt and drought (Zhao et al., 2020). In mulberry, ANK-type proteins enhance the chilling resistance by sustaining superoxide dismutase activity and unsaturated lipid content (Chen et al., 2018).

Exogenous combined application of SA and AA promotes the downregulation of core regulatory transcriptional factor MYC-like bHLH (basic helix–loop–helix) of the Jasmonic acid (JA) signaling mechanism, which binds to ICE1 afterwards, stimulates the expression of downstream CBF genes when subjected to low temperature conditions. In SA+AA combined-treated ornamental Chilli, ABA and JA might be as key upstream activators of the ICE-CBF/DREB1 signaling cascade, promoting COR gene expression, and upregulating COBRA, MYB, and ERF transcription. Our findings further demonstrate that the combined application of SA+AA appears to provoke SA signaling for stress-mitigating effects by positively activating the expression of the CBF downregulation gene ICE, and that downregulation of MYC, GRB, and ANK gene expression might represent an interaction between pathways of JA and SA, where SA+AA triggers the SA/JA-responsive genes set. Furthermore, the accumulation of ABA and JA was markedly increased in SA+AA-treated plants, which exhibited enhanced cold tolerance compared to non-treated plants. This suggests that the elevated levels of ABA and JA are potentially involved in the Chilling stress responses of ornamental chilli through the ICE-CBF-COR pathway. In conclusion, the mechanism behind Chilling stress in ornamental Chillies is an intricate process. Diverse pathways are engaged in molecular signaling regulation by chilling stress with foliar-sprayed SA and AA growth regulators in ornamental Chillies. Further studies are required to explain the molecular mechanisms behind the salicylic acid and ascorbic acid during Chilling stress in ornamental Chillies.

## Conclusion

**Figure 13:**
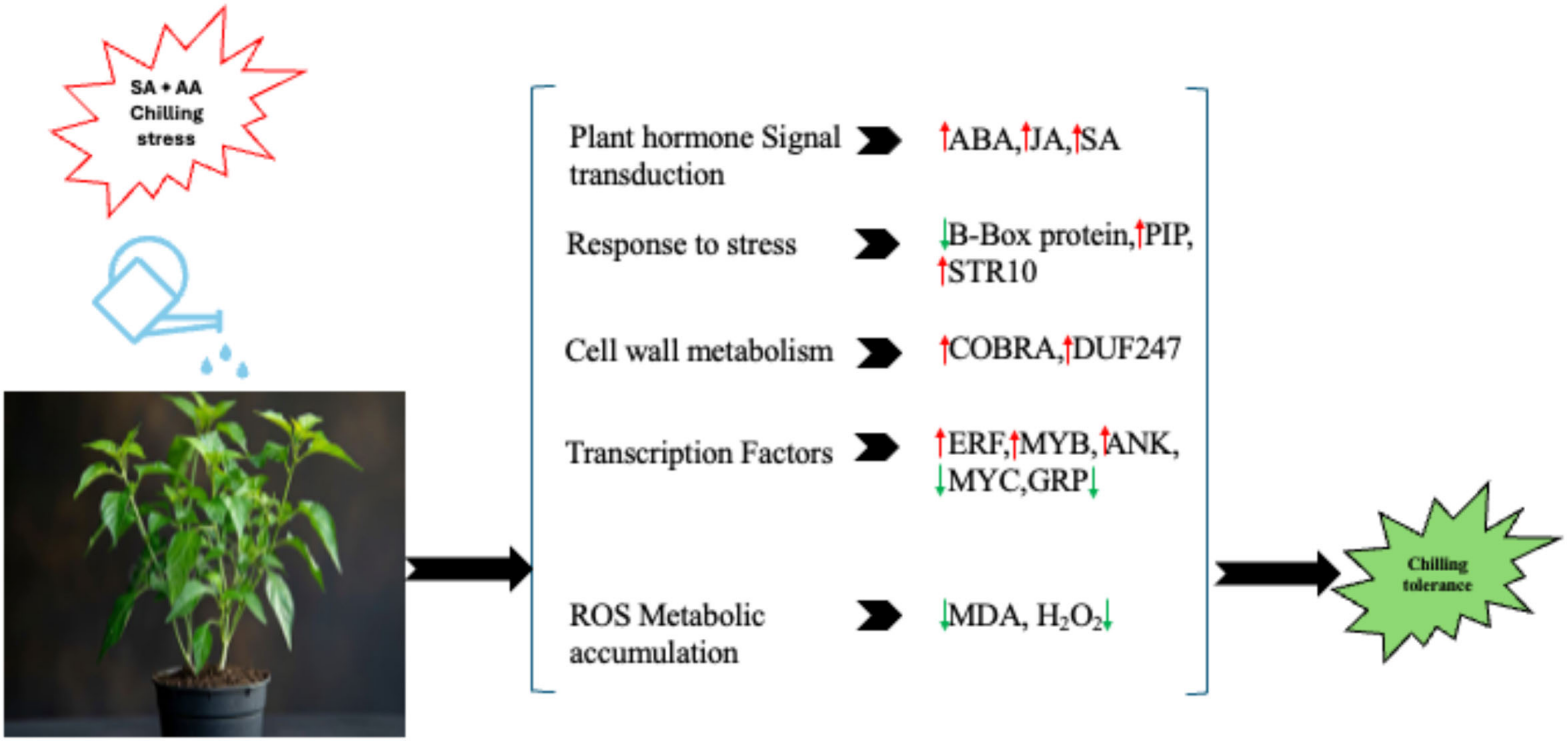
A Proposed model of salicylic acid (SA)-ascorbic acid (AA) enhancing chilling stress tolerance in ornamental Chillies. SA+AA increases the cold tolerance of the ornamental Chilli plants by reducing lipid peroxidation, promoting the expression of cell-wall metabolism genes, and other genes related to stress. Red arrows indicate upregulation, while green arrows indicate downregulation.

In this study, we demonstrated the molecular basis of SA + AA-enhanced chilling resistance in ornamental Chilli through transcriptomic analysis. Our findings indicate that SA + AA combined treatment intensified chilling tolerance in both cultivars, WIZ-21 and Golden Heart, through discrete transcriptional modulation. Gene ontology and KEGG pathway enrichment analyses highlighted that the combined foliar spray of SA + AA exposed to chilling stress strongly regulated genes associated with photosynthesis, the pentose phosphate pathway, sulfur metabolism, cellulose synthase-like proteins, as well as the biosynthesis of cysteine, amino acids, methionine, glyoxylate, dicarboxylate metabolism, and pyruvate metabolism. Additionally, the physiological function of each identified gene requires functional characterization using various methods, such as integrating multi-omics, single-cell, spatial transcriptomics, RNA velocity analyses, and long-read sequencing approaches. Moreover, we introduce a model representing the potential interplay between SA + AA treatment and core signaling pathways involving SA, ABA, and JA during SA + AA-stimulated cold tolerance (**Figure 13**). Overall, our findings confer the first transcriptional-level confirmation of the fundamental role of exogenous SA + AA in strengthening chilling tolerance in ornamental Chillies. The results highlight the modulation of the extensive signal transduction cascades, metabolic adjustments, and transcriptional networks that collectively influence adaptation to low-temperature stress. These implications not only advance our knowledge base of combined SA + AA-induced stress responses but also identify putative molecular targets for developing chilling stress-tolerant ornamental chilli cultivars through breeding or biotechnological strategies.

## Data Availability

The original contributions presented in the study are publicly available. This data can be found under accession number PRJNA1432074.
